# Circulating prostate cancer cells have differential resistance to fluid shear stress-induced cell death

**DOI:** 10.1242/jcs.251470

**Published:** 2021-02-22

**Authors:** Jacob M. Hope, Matthew R. Bersi, Jenna A. Dombroski, Andrea B. Clinch, Rebecca S. Pereles, W. David Merryman, Michael R. King

**Affiliations:** Department of Biomedical Engineering, Vanderbilt University, 5824 Stevenson Center, Nashville, TN 37235, USA

**Keywords:** Cancer, Mechanotransduction, Membrane

## Abstract

Circulating tumor cells (CTCs) are exposed to fluid shear stress (FSS) of greater than 1000 dyn/cm^2^ (100 Pa) in circulation. Normally, CTCs that are exposed to FSS of this magnitude die. However, some CTCs develop resistance to this FSS, allowing them to colonize distant organs. We explored how prostate CTCs can resist cell death in response to forces of this magnitude. The DU145, PC3 and LNCaP human prostate cancer cell lines were used to represent cells of different metastatic origins. The cell lines were briefly treated with an average FSS of 3950 dyn/cm^2^ (395 Pa) using a 30 G needle and a syringe pump. DU145 cells had no change in cell viability, PC3 cells had some cell death and LNCaP cells exhibited significant cell death. These cell death responses correlated with increased cell membrane damage, less efficient membrane repair and increased stiffness. Additionally, FSS treatment prevented the LNCaP FSS-sensitive cell line from forming a growing tumor *in vivo*. This suggests that these properties play a role in FSS resistance and could represent potential targets for disrupting blood-borne metastasis.

## Abstract (precis)

**Summary:** Prostate cancer cells have different sensitivities to fluid forces that alter their resistance to elevated blood flow-level fluid shear stress.

## INTRODUCTION

Metastasis accounts for ∼90% of cancer-related deaths (Seyfried and Huysentruyt, 2013). Cancer metastasis occurs when cancer cells detach from the primary tumor and invade the surrounding matrix. The cells then enter the circulation by intravasating through the endothelial cell wall. Once the cells enter the circulatory system, they are collectively referred to as circulating tumor cells (CTCs). After the CTCs disseminate throughout the body, they bind to endothelial cells on the vessel wall and extravasate into secondary sites. In the new site, the cancer cells proliferate to form a secondary tumor (Sahai, 2007; Valastyan and Weinberg, 2011; van Zijl et al., 2011).

CTCs have been associated with very negative prognoses for cancer patients due to their association with metastasis and the difficulty in targeting and treating the cells (Faltas, 2012; Mitchell et al., 2014; Ou et al., 2019). Despite the high morbidity of patients with CTCs, only a small subset (∼0.01%) of CTCs actually survive within the circulatory system (Cameron et al., 2000; Larson et al., 2004; Luzzi et al., 1998). The low survival rate of CTCs is due to the harsh physiological barriers present within the circulatory system. One of these barriers is the elevated fluid shear stresses (FSSs) present in the circulatory system (Mitchell and King, 2013a; Regmi et al., 2017). The FSS of the circulatory system normally ranges from 0.5 to 4.0 dyn/cm^2^ (0.05­–0.4 Pa) in the venous compartment and from 4.0 to 30.0 dyn/cm^2^ (0.4–3.0 Pa) within the arterial system. However, at regions of arterial bifurcation or within the heart, CTCs can briefly experience greater than 1000 dyn/cm^2^ (100 Pa) of FSS (Huang et al., 2018).

The mechanical mechanisms hypothesis states that the pattern of blood flow that a CTC experiences determines whether a CTC can successfully colonize a distant organ (Liu et al., 2017). By this hypothesis, a CTC must be resistant to the FSS it will experience when traveling to the distant site. For a prostate CTC to colonize the brain, it will have to be resistant to the greater than 1000 dyn/cm^2^ (100 Pa) FSS it will experience when passing through the heart. Normally, these elevated FSSs induce cell death by causing physical damage to the CTCs (Moose et al., 2020). This damage can cause holes to form in the cell membrane. If these holes are not quickly patched, cell death can occur as the holes allow for uncontrolled ion flux, ATP flux, and even organelle flux between the intracellular and extracellular environments (Cooper and McNeil, 2015; Idone et al., 2008; Reddy et al., 2001). Therefore, it is likely that metastatic cancer cells evolve mechanisms to resist FSS by preventing membrane damage or by efficiently repairing the membrane.

A previous study identified that PC3 prostate cells have an innate resistance to cell death by FSS in comparison to healthy prostate epithelial cells (Barnes et al., 2012). Other studies have established proteins that are necessary for CTC survival in elevated FSS conditions, such as RhoA for efficient membrane repair (Moose et al., 2020). Additionally, our lab identified lamin A/C as necessary for FSS survival in CTCs by ensuring nuclear membrane integrity (Mitchell et al., 2015). The aim of the current study was to compare the differential cell death responses of prostate cancer cells to FSS, and to determine whether characteristics such as membrane damage and stiffness govern these responses. This was accomplished by treating cells with brief pulses (∼1 ms) of FSS that averaged 3950 dyn/cm^2^ (395 Pa) using a syringe pump-based apparatus. This level of FSS was selected because a previous study identified this magnitude of FSS as representing a physiologically relevant level at which healthy epithelial cells were sensitive to FSS for these brief pulses, whereas PC3 cancer cells were resistant (Barnes et al., 2012). Additionally, this elevated FSS was studied in favor of lower FSSs because previous studies have looked at the effect of lower FSSs on CTC cell death (Hope et al., 2019, 2018; Huang et al., 2018; Lien et al., 2013; Mitchell and King, 2013b; Regmi et al., 2017). The short pulse duration was also selected because CTCs will primarily experience such elevated FSS for short durations on the order of milliseconds (Strony et al., 1993). Three prostate cancer cell lines were selected based on the different metastatic locations from which they were derived, because we predicted that their different metastatic origins may be suggestive of different FSS resistances. The DU145 cell line was isolated from a brain metastasis, where it would have had to pass through the heart and the carotid bifurcation, thus experiencing greater than 1000 dyn/cm^2^ of FSS (Cheon and Chandran, 1993; Yin et al., 2010; Malek et al., 1999; Strony et al., 1993). The highly metastatic PC3 line was also selected and was isolated from the bone (Chu et al., 2008). For the PC3 cells to metastasize to the bone, they would have been exposed to approximately 10 dyn/cm^2^ (1 Pa) of FSS (Gray and Stroka, 2017). Lastly, the lymph node metastatic-derived LNCaP cell line was chosen (Castanares et al., 2016). Due to the significantly lower FSS within the lymphatic system, the LNCaP cells would likely have been exposed to the lowest magnitude of FSS of the three cell lines (Kornuta et al., 2015). In this study, we determined that prostate cancer cell lines show differential sensitivities to FSS-induced cell death and that the FSS sensitivity is consistent with their degree of membrane damage, membrane repair and their biophysical properties, such as cell stiffness and fluidity. An animal model was also utilized to determine whether DU145 and LNCaP cells could successfully form a growing tumor after FSS exposure.

## RESULTS

### FSS treatment induces cell death in PC3 and LNCaP cells

**Fig. 1.**
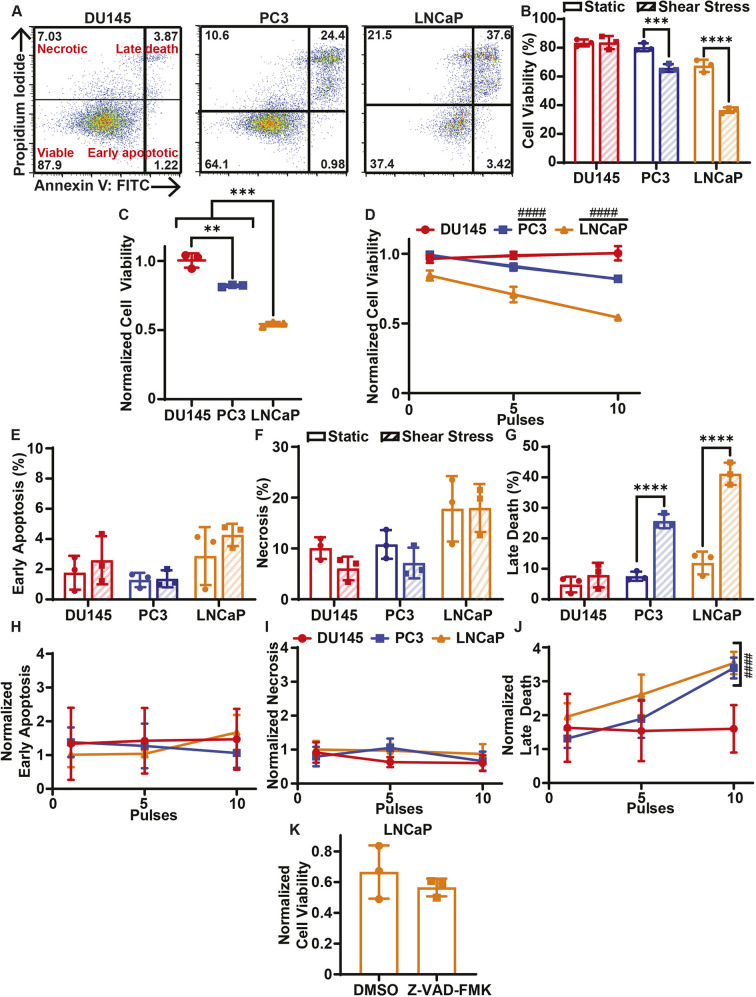
**Cell death of cancer cells treated with elevated FSS.** (A) Representative AV/PI flow cytometry scatter plots of DU145, PC3 and LNCaP cells. The percentage of the cell population in each cell gate is indicated on the plots. (B) Average cell viabilities of cancer cells treated with (shear stress) or without (static) 10 pulses of FSS. (C) Average cell viabilities of cancer cells treated with FSS normalized to untreated static controls. (D) Average normalized cell viabilities of cancer cells as a function of the number of FSS pulses, ranging from 0 to 10. (E) Average early apoptotic, (F) necrotic and (G) late apoptotic populations for cancer cells treated with 0 or 10 pulses of FSS. (H) Average early apoptotic, (I) necrotic and (J) late apoptotic populations of cancer cells as a function of FSS pulses, normalized to the static control. (K) Cell viability of LNCaP cancer cells treated with 50 µM Z-VAD-FMK or DMSO prior to FSS treatment, normalized to static controls. Data are presented as mean±s.d. *N*=3 independent experiments. **P<0.01, ***P<0.005, ****P<0.001 (unpaired two-tailed *t*-test). ^####^*P*<0.001 (least squares linear regression to confirm significant deviation from zero).

To determine whether the different prostate cancer cells exhibit different sensitivities to FSS, the cancer cells were treated with 0–10 pulses of FSS. The average FSS of each pulse was 3950 dyn/cm^2^ (395 Pa) for a duration of 1.08 ms. Cell viability was measured 24 h following FSS treatment using the annexin V/propidium iodide (AV/PI) assay (Fig. 1A; Fig. S1A). DU145 cells treated with 10 pulses of FSS showed no reduction in cell viability 24 h after FSS treatment (Fig. 1B). PC3 and LNCaP cells, however, experienced significant reductions in cell viability after treatment with 10 pulses of FSS (Fig. 1B). The cell viability reduction of the LNCaP cells was more pronounced than it was for the PC3 cells. This trend was further seen when the FSS-treated cells were normalized to their static controls, with DU145 cells being the most viable, PC3 cells being the second most viable and the LNCaP cells being the least viable (Fig. 1C). This suggests that the prostate cancer cell lines have different sensitivities to elevated FSS. The effect of varying the amount of FSS on cell death was assessed by treating the cancer cells with 1, 5 and 10 pulses of FSS and normalizing the cell viabilities to the respective static untreated control (Fig. 1D). The DU145 cells showed no change in cell viability despite increasing the number of FSS pulses. For both the PC3 and LNCaP cell lines, increasing the number of FSS pulses further reduced cell viability, as the best-fit slope yielded by least-squares linear regression significantly deviated from zero (Fig. 1D).

The AV/PI assay was also used to identify the form of cell death the cancer cells were undergoing in response to FSS. For the DU145 cells, there was no significant increase in the necrotic, early apoptotic or late apoptotic populations following 24 h of FSS exposure (Fig. 1E–G). Varying the amount of FSS exposure also did not alter the necrotic, early-stage apoptotic and late apoptotic population fractions for DU145 cells (Fig. 1H–J). The LNCaP and PC3 cells did not show significant increases in the early apoptotic or the necrotic cell populations when treated with 10 pulses of FSS compared to their static controls (Fig. 1E,F). Likewise, these population fractions did not change in response to the amount of FSS treatment (Fig. 1H,I). The LNCaP and PC3 cells both showed a significant increase in their late apoptotic populations following 10 pulses of FSS (Fig. 1G). The late apoptotic populations significantly increased in correspondence with increasing amounts of FSS treatment (Fig. 1J). To determine whether the cell death was apoptotic or necrotic in nature, LNCaP cells were pretreated with the pan-caspase inhibitor Z-VAD-FMK 1 h prior to FSS treatment. Z-VAD-FMK did not significantly increase cell viability in response to FSS treatment, suggesting that the cell death was necrotic rather than apoptotic (Fig. 1K).

### FSS treatment causes differential cell membrane damage among prostate cancer cell lines

**Fig. 2.**
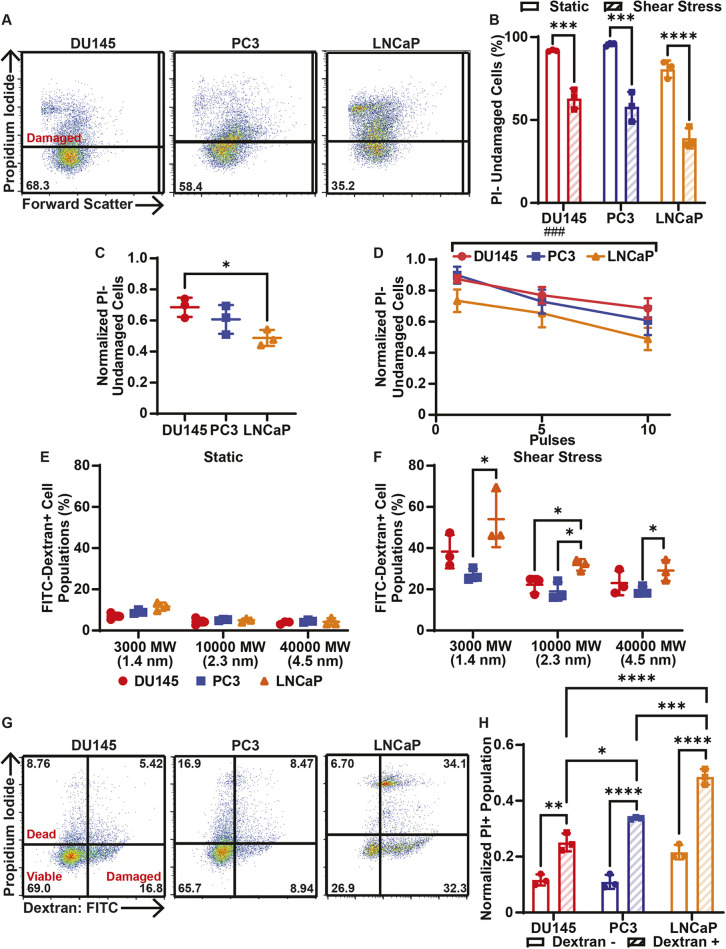
**Cell damage and related cell death of cancer cells treated with elevated FSS.** (A) Representative PI flow cytometry scatter plots to quantify cell membrane damage in DU145, PC3 and LNCaP cells. The percentage of the cell population in the undamaged cell gate (PI negative) is shown for each plot. (B) Percentage of undamaged cancer cells in populations treated with (shear stress) or without (static) 10 pulses of FSS. (C) Average number of PI-negative (undamaged) cancer cells treated with FSS, normalized to untreated static controls. (D) Normalized undamaged cancer cells as a function of FSS pulses, ranging from 0 to 10. (E) Uptake of FITC-tagged dextrans of different MW and hydrodynamic radii by cancer cells under static conditions as a measure of cell membrane damage. (F) Uptake of different dextrans by cancer cells treated with 10 pulses of FSS. Cell lines are color-coded as in E. (G) Representative flow cytometry scatter plots of 3000 MW FITC-tagged dextran/PI cell fate tracking. Cells positive for dextran indicate cell membrane damage, cells positive for PI represent dead cells, and cells negative for both indicate viable cells. The percentage of the cell population in each cell gate is indicated on the plots. (H) Proportion of PI-positive cells, normalized to to the corresponding undamaged dextran subpopulation, in dextran-negative and dextran-positive populations of cancer cells treated with 10 pulses of FSS. Data are presented as mean±s.d. *N*=3 independent experiments. *P<0.05, **P<0.01, ***P<0.005, ****P<0.001 (unpaired two-tailed *t*-test). ^###^*P*<0.005 (simple linear regression to confirm significant deviation from zero).

Elevated FSS is known to cause cell membrane damage that can result in cell death due to uncontrolled ion flux and ATP leakage (Dong et al., 2006; Horn and Jaiswal, 2018; Moose et al., 2020). DU145, PC3 and LNCaP cells were incubated with PI before and during FSS treatment to measure cell membrane damage by FSS (Fig. 2A; Fig. S1B). PI cannot pass through an intact cell membrane. Therefore, observation of PI fluorescence following FSS treatment is indicative of cell membrane permeabilization. Each cancer cell line exhibited a significant reduction in the number of undamaged cells after 10 pulses of FSS treatment (Fig. 2B). The number of undamaged cells after 10 pulses of FSS treatment was normalized to the number of undamaged cells with no FSS treatment. DU145 cells had a significantly greater normalized undamaged cell population in comparison to LNCaP cells, indicating that DU145 cells suffered less damage from FSS treatment (Fig. 2C). For each cancer cell line, increasing numbers of FSS pulses caused increasingly significant reductions in the normalized population of undamaged cells (Fig. 2D).

To quantify the magnitude of the perforations forming in DU145, PC3 and LNCaP cell membranes due to FSS, the cells were incubated with fluorescent dextrans of different molecular weights (3000, 10,000 and 40,000 MW) and corresponding hydrodynamic radii prior to, and during, FSS treatment (Fig. 2E,F). For the untreated static conditions, there was no significant difference between any of the three cell lines in 3000 MW, 10,000 MW or 40,000 MW dextran uptake (Fig. 2E). However, for the cells treated with 10 pulses of FSS, the LNCaP cells absorbed more 3000 MW and 40,000 MW dextran than both the PC3 and DU145 cells. However, the increased uptake was only determined to be significant when comparing the PC3 and LNCaP cell lines for these two dextrans. For the 10,000 MW dextran, LNCaP cells absorbed significantly more than both the DU145 and PC3 cells (Fig. 2F). These results further suggest that LNCaP cells suffer more membrane damage in response to FSS exposure.

To determine whether the cells that suffered cell membrane damage were more likely to undergo cell death, cancer cells were incubated with 3000 MW dextran before and during 10 pulses of FSS treatment. At 24 h after FSS treatment, cell death was measured using PI. Cells that were only positive for 3000 MW dextran were considered to be damaged but viable. Cells that were only positive for PI were considered dead but undamaged. Cells positive for both PI and dextran were considered to be dead and damaged (Fig. 2G; Fig. S1C). For all three cell lines, the proportion of PI-positive cells was significantly increased for dextran-positive cells. This indicates that cells damaged by FSS are more likely to undergo cell death by FSS treatment compared to undamaged cells. LNCaP cells that were damaged had a significantly greater proportion of PI-positive cells than the damaged PC3 and DU145 cells, again indicating that LNCaP cells are more sensitive to FSS-induced cell death (Fig. 2H).

### Cell membrane repair efficiency correlates with FSS resistance

**Fig. 3.**
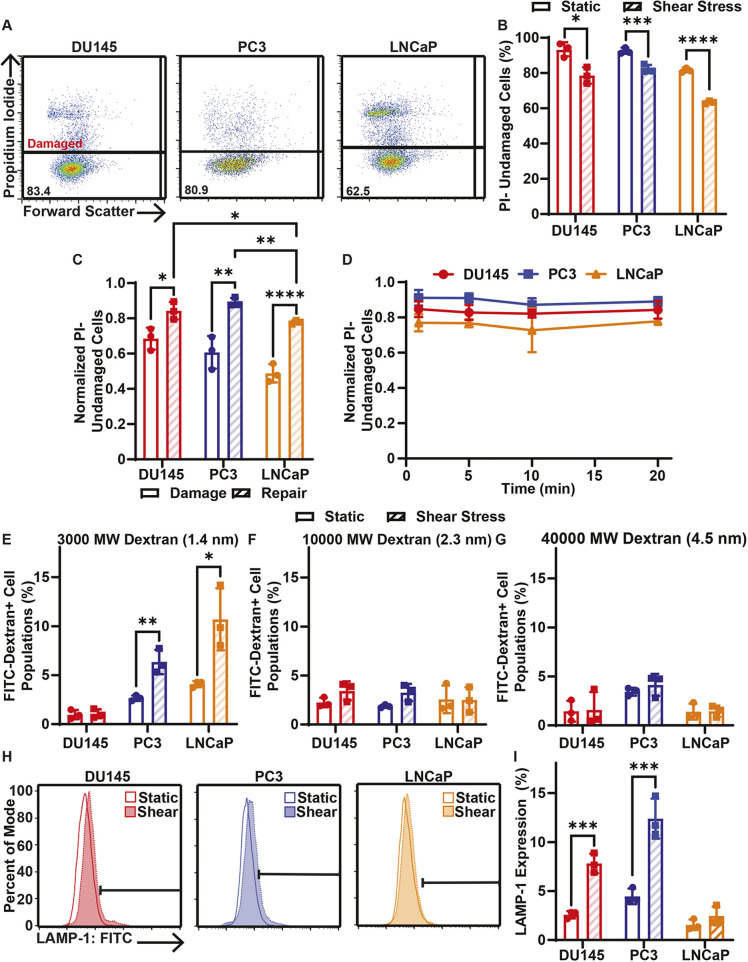
**Cell membrane repair of cancer cells treated with 10 pulses of elevated FSS.** (A) Representative PI flow cytometry scatter plots of membrane damage for DU145, PC3 and LNCaP cells after allowing 20 min of time for cell membrane repair. The percentage of the cell population in the undamaged cell gate (PI negative) is shown for each plot. (B) Average percentage of cancer cells treated with (shear stress) or without (static) 10 pulses of FSS that were undamaged after 20 min of cell membrane repair time. (C) Average proportion of undamaged cells for cell membrane damage measurements taken during FSS (damage) and for cells provided with 20 min of repair time after FSS (repair). (D) Normalized PI-negative (undamaged) cancer cells after different cell membrane repair times, ranging from 1 to 20 min. (E) Uptake of FITC-tagged 3000 MW, (F) 10,000 MW and (G) 40,000 MW dextran by cancer cells treated with or without 10 FSS pulses following 20 min of cell membrane repair. (H) Representative flow cytometry histograms of surface LAMP-1 staining. Horizontal bar indicates the LAMP-1-positive cell gate. (I) Average surface LAMP-1 expression for cancer cells treated with (hatched bars) or without (open bars) 10 pulses of FSS. Data are presented as mean±s.d. *N*=3 independent experiments. *P<0.05, **P<0.01, ***P<0.005, ****P<0.001 (unpaired two-tailed *t*-test). In D, least squares linear regression showed no significant deviation from zero.

If cell membrane damage is not rapidly repaired, further cell death can ensue (Howard et al., 2011). To determine whether the cell membrane damage caused by FSS treatment is sustained, cell membrane damage was measured 20 min after 10 pulses of FSS treatment by incubating the cells with PI 20 min after FSS treatment (Fig. 3A; Fig. S1B). The 20 min period was chosen as a sufficient amount of time for repair to take place because membrane repair in normal circumstances is a rapid process occurring in seconds to minutes (Tang and Marshall, 2017). There was still a significant reduction in the undamaged cell population for the 20 min post-FSS treatment group in comparison to the static control for each cell line (Fig. 3B). The undamaged cell populations after 20 min of FSS exposure for each cell line were normalized to their untreated controls. When these normalized repair populations were compared to the normalized population of cells damaged during FSS treatment, there was a significant increase in the normalized undamaged cell populations for the repair condition in each cell line (Fig. 3C). These results together suggest that while some of the cancer cells from each cell line are permanently damaged by FSS, repair does take place for many of these damaged cells. When comparing the effect of the 20 min repair condition on each cell line, DU145 and PC3 cells had significantly higher normalized undamaged cell populations than the LNCaP cells, indicating that LNCaP cells suffered more permanent damage by FSS (Fig. 3C). To assess whether each cell line had a different rate of repair, cell membrane damage was measured from 1 to 20 min following 10 pulses of FSS treatment. For each cell line there was no major change in the undamaged cell population, even when comparing the 1 and 20 min groups (Fig. 3D). This suggests that, for cells that undergo membrane repair, the membrane repair occurs quickly for each cell line with respect to PI permeability.

Membrane repair was also measured using dextran uptake 20 min post-FSS treatment (Fig. 3E–G). For 3000 MW dextran, DU145 cells did not show a significant increase in dextran fluorescence when comparing the shear-treated group to the static control, indicating that membrane repair was efficient for DU145 cells. PC3 and LNCaP cells showed significantly increased dextran fluorescence 20 min after FSS treatment compared with that of their respective untreated control groups (Fig. 3E). This suggests that membrane repair may not be as efficient for PC3 and LNCaP cells. When measuring repair for 10,000 and 40,000 MW dextrans, there was no significant increase in dextran fluorescence for all cell lines, meaning that these larger membrane damage events were at least partially healed 20 min following FSS treatment (Fig. 3F,G).

Finally, surface lysosomal associated membrane protein-1 (LAMP-1) was used to identify cell membrane repair after FSS exposure, because LAMP-1 is known to translocate to the membrane surface for membrane repair (Reddy et al., 2001). Cancer cells were live stained with an anti-LAMP-1 antibody 5 min after FSS treatment for 30 min (Fig. 3H; Fig. S1D). DU145 and PC3 cells showed a significant increase in surface LAMP-1 expression after FSS treatment, whereas LNCaP cells showed no change in LAMP-1 (Fig. 3I). This again indicates that membrane repair is not as efficient in the LNCaP cells.

### Resistance to FSS-induced apoptosis correlates with increased cell stiffness and reduced cell fluidity

**Fig. 4.**
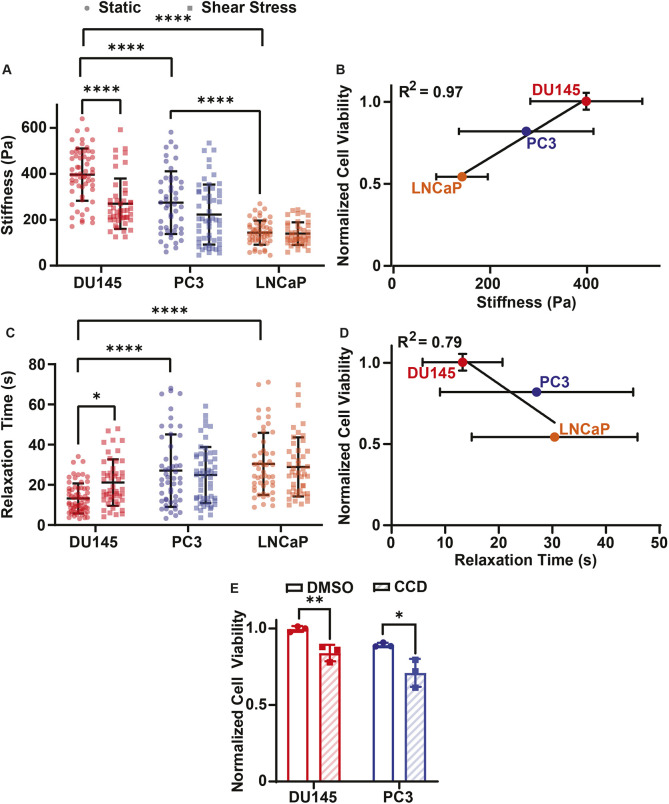
**Correlation of biophysical properties and prostate cancer cell viability following FSS exposure.** (A) Stiffness of DU145 (static, *n*=58; shear, *n*=46), PC3 (static, *n*=47; shear, *n*=54) and LNCaP (static, *n*=51; shear, *n*=41) cells before (static) and after (shear stress) 10 pulses of FSS. (B) Correlation between normalized cell viability and cell stiffness. (C) Relaxation time of DU145 (static, *n*=65; shear, *n*=48), PC3 (static, *n*=46; shear, *n*=54) and LNCaP (static, *n*=52; shear, *n*=45) cells before and after FSS exposure, as in A. (D) Correlation between normalized cell viability and relaxation time. (E) Cell viability of DU145 and PC3 cells pretreated with DMSO or 20 µM CCD and then treated with 10 pulses of FSS. Viability of FSS-treated cells was normalized to static conditions. Data are presented as mean±s.d. Measurements from *N*=3 independent experiments of stiffness and relaxation time. *N*=3 independent experiments for normalized cell viability. *P<0.05, **P<0.01, ****P<0.001 (unpaired two-tailed *t*-test). Linear regression was used to calculate R^2^ in B and D.

Increased cell stiffness has previously been associated with resistance to cell death induced by elevated FSS (Barnes et al., 2012; Chivukula et al., 2015). To determine whether mechanical stiffness correlated with resistance to FSS-induced cell death, the stiffness of each cancer cell line treated with or without FSS was measured using micropipette aspiration (Taneja et al., 2020). Micropipette aspiration was also used to assess cell fluidity by measuring the viscoelastic relaxation time of the cells. Prior to FSS treatment, DU145 cells were found to be significantly stiffer than both the LNCaP and PC3 cell lines. The PC3 cells were also significantly stiffer than the LNCaP prior to FSS exposure (Fig. 4A). When the LNCaP and PC3 cells were treated with 10 pulses of FSS, no significant change in stiffness was observed in comparison to the stiffness of untreated cells. The DU145 cells showed a significant reduction in stiffness following exposure to FSS when compared to their non-sheared controls, while also displaying a significantly greater change in stiffness compared to those of PC3 and LNCaP cells (Fig. 4A; Fig. S2A). Normalized cell viability strongly correlated with increased cell stiffness, with an R^2^ value of 0.97 as calculated by linear regression (Fig. 4B). Additionally, the viscoelastic relaxation time of DU145 cells was significantly lower than that of the PC3 and LNCaP cells, indicating that DU145 cells have a less fluid-like phenotype. Furthermore, only the DU145 cells exhibited a significant change in relaxation time following FSS treatment (Fig. 4C; Fig. S2B). Relaxation time and normalized cell viability had a negative correlation, with an R^2^ of 0.79, suggesting that reduced cell fluidity may impart FSS resistance as well (Fig. 4D).

To further study the effects of these mechanical properties on the FSS resistance of cancer cells, DU145 and PC3 cells were pretreated with 20 µM cytochalasin D (CCD) 1 h prior to FSS exposure, because CCD has previously been shown to significantly reduce cell stiffness (Rotsch and Radmacher, 2000). Modifying stiffness with CCD was found to significantly reduce the normalized cell viability of FSS-treated cells compared with that of the DMSO control group for both PC3 and DU145 cells (Fig. 4E).

### FSS treatment prevents successful *in vivo* tumor growth

**Fig. 5.**
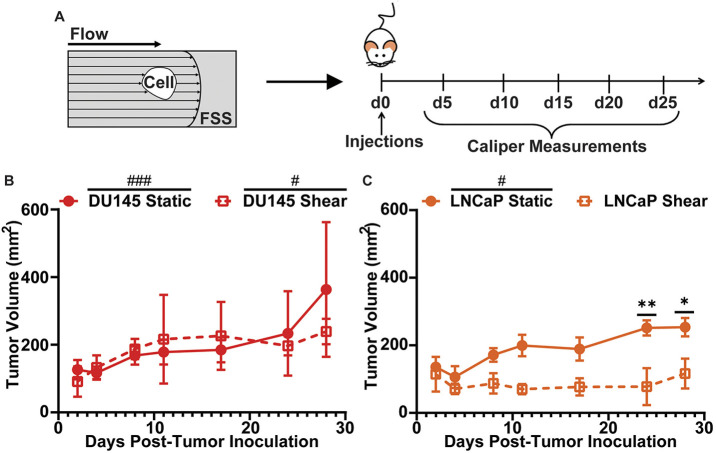
***In vivo* mouse model of tumor growth of FSS-treated and untreated cancer cells.** (A) Schematic of the mouse experiment. DU145 and LNCaP cells were treated with or without 10 pulses of FSS and then subcutaneously injected into mice. Tumor growth was measured using calipers over a 28-day period. (B) Tumor growth of DU145 cells treated with (shear) or without (static) FSS and of (C) LNCaP cells treated with or without FSS.  Data are presented as mean±s.d. *N*=3 independent experiments. *P<0.05, **P<0.01 (unpaired two-tailed *t*-test used to compare groups). ^#^*P*<0.05, ^###^*P*<0.005 (simple linear regression to confirm significant deviation from zero).

To determine whether FSS exposure can hinder a cancer cell's ability to colonize and form a growing tumor at a distant location, DU145 and LNCaP cells were treated with or without 10 pulses of FSS before being subcutaneously implanted into mice. After implantation, tumor volume was monitored over 28 days using calipers (Fig. 5A). The 10 pulses of FSS were used to roughly model the elevated FSS CTCs would experience in colonizing a distant site, such as the brain. Thus, a subcutaneous tumor was used to approximate a ‘secondary’ tumor site, with tumor growth being used as a proxy for viability. DU145 and LNCaP cells were used in this mouse experiment to focus on both the most FSS-sensitive and most FSS-resistant cells. Both sheared and non-sheared DU145 cells demonstrated significant tumor volume growth over the 28 days, with linear regression analysis showing a slope that significantly deviated from zero. There was also no significant difference in tumor volume between the static and sheared DU145 cells at any point over the 28 days (Fig. 5B). The LNCaP static control cells showed significant tumor volume growth over 28 days, whereas the LNCaP shear-treated cells did not have significant tumor volume growth. Also, at days 24 and 28 there was a significant difference in tumor volume between the static and sheared LNCaP cells (Fig. 5C). These results demonstrate that FSS exposure can prevent FSS-sensitive cells, such as the LNCaP cells, from forming a healthy growing tumor. The FSS-resistant DU145 cells, however, were unaffected by FSS exposure and formed a healthy tumor despite the FSS treatment. This observation is consistent with the *in vitro* results where the DU145 cell viability was unaffected by FSS treatment, whereas LNCaP cells had a dramatic reduction in viability.

## DISCUSSION

A previous study determined that the PC3 prostate cancer cell lines are innately more resistant to FSS than healthy prostate epithelial cells, suggesting that FSS resistance contributes to metastasis (Barnes et al., 2012). The results of the present study show that both DU145 and PC3 cells exhibit at least some resistance to FSS, whereas the LNCaP cells were found to be quite sensitive to FSS (Fig. 1A–D). This suggests that resistance to FSS is not a conserved property in all metastatic prostate cancer cells. Previous studies have shown that elevated FSS can contribute to apoptosis and necrosis in CTCs (Mitchell et al., 2015; Moose et al., 2020). The mode of cell death in response to FSS was measured in this study as well for each of the cell lines. The three cell lines in this study had no increase in the necrotic or early-stage apoptosis populations when treated with 3950 dyn/cm^2^ (395 Pa) of FSS. However, the PC3 and LNCaP cells showed a significant increase in the late apoptotic populations (Fig. 1E–J). When pretreating LNCaP cells with Z-VAD-FMK, there was no reduction in their normalized viability, which indicates that FSS in these experiments induced necrotic cell death in the case of LNCaP cells (Fig. 1K).

Elevated FSS is known to cause deformations and permeabilize the plasma membrane of cancer cells, resulting in cell death (Moose et al., 2020; Rejniak, 2016, 2012). Each prostate cancer cell line suffered significant cell membrane damage upon FSS treatment (Fig. 2A–D). The use of different molecular weight dextrans allowed us to further characterize the scale of cell membrane damage events caused by FSS in each cell line. LNCaP cells experienced more extensive damage than PC3 and DU145 cells, as the LNCaP cells showed increased uptake of dextrans of all MW. This implies a correlation between cell membrane damage by FSS and cell death by FSS (Fig. 2E,F). To further establish the link between membrane damage and cell death, all three cancer cell lines were dually stained with 3000 MW dextran and PI, where dextran fluorescence indicates membrane damage and PI fluorescence indicates cell death (Fig. 2G). For each cancer cell line, positive dextran staining correlated with a significant increase in the proportion of PI-positive cells, supporting the claim that membrane damage contributes to cell death caused by FSS. LNCaP cells also showed a significant increase in the normalized PI-positive population of dextran-positive cells, suggesting that LNCaP cells that suffered from membrane damage are more likely to undergo cell death by FSS (Fig. 2H).

Previous studies established that cell membrane repair is necessary for survival when the membrane is compromised (Horn and Jaiswal, 2018; Li et al., 2014; Moose et al., 2020). It has also been found that proteins associated with cell membrane repair are upregulated in metastatic cancer cells (Jaiswal et al., 2014). Because each of the cell types we studied are metastatic to some degree, it was not unexpected that each cell line would see significant reductions in membrane damage when time was allowed for membrane repair following FSS exposure (Fig. 3A–D). However, it is notable that not all cell lines were equally efficient in cell membrane repair. As measured using PI, LNCaP cells had significantly fewer undamaged cells after repair compared to PC3 and DU145 cells (Fig. 3C). When comparing static and FSS-treated cells for all three cancer cell lines, there was no increased uptake of 10,000 and 40,000 MW dextran after time was allowed for membrane repair (Fig. 3F,G). This means that the more extensive damage events suffered by each cell during FSS were at least partially healed. However, PC3 and LNCaP cells showed significantly greater uptake of 3000 MW dextran, while DU145 cells did not (Fig. 3E). When measuring membrane repair using surface LAMP-1 expression, both DU145 and PC3 cells exhibited significant increase in LAMP-1 expression, whereas LNCaP cells did not (Fig. 3H,I). The cell membrane repair efficiency as measured by these assays correlated with how well a cancer cell line resisted FSS-induced cell death, with LNCaP cells being the most sensitive to FSS and being the least efficient at membrane repair, DU145 cells having the most efficient repair and suffering the least amount of death, and PC3 cells being intermediate compared to the other two cell lines.

This study also established that stiffness correlates with FSS resistance and that fluidity has a negative correlation with FSS resistance, perhaps by reducing the magnitude of membrane damage that requires repair (Fig. 4A–D). A previous study identified PC3 cells as being stiffer than healthy prostate epithelial cells, suggesting that increased stiffness in prostate cancer cells supports a more metastatic phenotype (Chivukula et al., 2015). This is consistent with our observations. The stiffer DU145 and PC3 cells were more resistant to FSS-induced cell death than the softer LNCaP cells (Fig. 4A,B). This role of stiffness in FSS resistance was further supported by the effects of pretreating PC3 and DU145 cells with CCD, which is known to reduce cell stiffness (Rotsch and Radmacher, 2000). LNCaP cells were not tested with CCD as they were previously found to be sensitive to FSS and the aim of the CCD treatment was to determine whether it could make FSS-resistant cells sensitive to FSS. When PC3 and DU145 cells were treated with CCD, there was a significant reduction in normalized viability for cells also exposed to FSS (Fig. 4E). Likewise, a previous study has shown that reduced expression of lamin A/C, a protein known to promote cell stiffness, also has the effect of sensitizing cancer cells to FSS (Lee et al., 2007; Mitchell et al., 2015). In contrast to the findings of this study, increased fluidity and reduced stiffness traditionally correlate with more aggressive cancer metastasis (Khan et al., 2018). The reduced stiffness of cancer cells has been found to promote invasion and migration by allowing cancer cells to pass through confinements in the tumor microenvironment (Wullkopf et al., 2018). With respect to the current study, PC3 cells are well known to be strongly metastatic, DU145 cells are considered to be moderately metastatic and LNCaP cells to be weakly metastatic (Ravenna et al., 2014). This suggests that there may be an optimal cell stiffness for enhanced metastasis. A certain degree of cancer cell stiffness may allow a cancer cell to sufficiently navigate confined spaces and impart enough resistance to FSS to form a secondary metastatic tumor.

It was found that DU145 cells underwent a form of mechano-adaptation as they became softer and more fluid-like in response to FSS treatment (Fig. S2). As treating PC3 and DU145 cells with CCD increased the cytotoxicity of FSS, this softening of DU145 cells likely does not promote FSS resistance (Fig. 4E). Instead, the mechano-adaptation may promote the ability of DU145 cells to more efficiently extravasate and invade a secondary tumor site, since softer cells are associated with the ability to better navigate confined spaces (Wullkopf et al., 2018). The reduced stiffness of FSS-treated DU145 cells was unexpected, because a previous study that used the same method of FSS treatment found that PC3 cells become stiffer after FSS treatment (Chivukula et al., 2015). However, FSS exposure has also previously been shown to reduce cell stiffness (Xin et al., 2019). One possibility for this discrepancy is the divergence of cells cultured in different labs for different lengths of time. Another explanation for the results of this study might be that the stiffness was immediately analyzed following FSS exposure. Cell membrane repair traditionally requires cytoskeleton disassembly to relieve tension in the cell, which reduces the force needed for membrane wound closure (Abreu-Blanco et al., 2012). This cytoskeletal disassembly causes cell softening (Kasas et al., 2005). However, at later time points in membrane repair, these cytoskeleton proteins are replaced, possibly causing the increased stiffness observed in the previous study (Abreu-Blanco et al., 2012; Chivukula et al., 2015).

It is interesting that the resistance to elevated FSS correlates with the FSS the cancer cells would have experienced during their transit to the metastatic location from which they were derived. This aligns with the mechanical mechanisms hypothesis of cancer metastasis, which states that the pattern of blood flow determines whether a cancer cell can successfully colonize a distant organ (Liu et al., 2017). The DU145 cells, which are derived from a brain metastasis and thus have successfully passed through the elevated FSS environment of the heart in the patient of origin, demonstrate the FSS resistance necessary to survive such a trip. Likewise, PC3 cells, derived from a bone metastasis and thus subjected to FSS of the peripheral circulation (but perhaps not the heart), now exhibit a moderate FSS resistance consistent with their metastatic path. LNCaP cells, derived from a lymph node metastasis and likely to have never experienced blood circulatory FSS in the original patient, exhibit the weakest FSS resistance, which is consistent with their pathological history. This conclusion is further supported by the mouse experiments performed in this study, where the FSS-resistant DU145 cells treated with FSS prior to subcutaneous implantation in mice successfully formed viable growing tumors. The FSS-treated LNCaP cells did not form tumors that showed significant growth over the 28 day period (Fig. 5).

Traditionally, orthotopic and intravenous injection models have been used to study cancer cell metastasis, as they faithfully recreate multiple complex steps in the metastatic cascade. The subcutaneous model of this study was used instead because the other model systems involve aspects of metastasis that cannot be easily controlled. For example, in orthotopic or injection models, the cancer cells would be exposed to varying amounts of FSS for different durations. This could potentially overshadow the effect of the 10 pulses of elevated FSS that we aimed to study. The orthotopic and injection methods also involve measuring the formation of metastatic lesions, which can also be affected by variables unrelated to FSS, such as reduced cell viability by cancer cell constriction within capillaries or the suitability of a specific site for secondary tumor colonization (Nath et al., 2018). In effect, the subcutaneous model used in this study isolates the role of elevated FSS and more directly ascertains its effect on secondary tumor site colonization. However, this method is simplistic compared to the complex pathway of cancer metastasis, and so represents a partial picture. Despite this, the *in vivo* results of this study are consistent with previous studies that use intravenous injection to measure the metastatic ability of DU145 and LNCaP cells. DU145 cells, when intravenously injected, were able to form metastatic lesions (Chen et al., 2014; Sun et al., 2008; Teng et al., 2010). LNCaP cells, however, have been shown to be unable to form metastatic lesions when injected into mice through tail vein injection (Steffan et al., 2012; Wu et al., 1998).

It is important to note that these cancer cell lines were derived from different patients. Thus, there is significant genetic heterogeneity among these cell lines. To more conclusively identify if properties such as stiffness promote FSS resistance, it would be interesting to determine whether cancer cell lines of different metastatic potentials that are derived from the same patient have similar results to those presented in this study. However, the findings of this study point to possible therapeutic strategies for targeting CTCs to prevent blood-borne metastasis. Pharmacological tools could be used to further exacerbate the cell membrane damage by promoting CTC deformability, or by undermining cell membrane repair (Mitchell et al., 2015; Moose et al., 2020; Ortiz-Otero et al., 2020).

## MATERIALS AND METHODS

### Cell culture

Prostate adenocarcinoma cell lines PC3 (ATCC #CRL-1435), DU145 (ATCC #HTB-81) and prostate carcinoma cell line LNCaP (ATCC #CRL-1740), were purchased from American Type Culture Collection (Manassas, VA, USA). PC3, DU145 and LNCaP cells were cultured in RPMI 1640 cell culture medium (Invitrogen, Grand Island, NY, USA). Medium was supplemented with 10% (v/v) fetal bovine serum and 1% (v/v) PenStrep, all purchased from Invitrogen. PC3, DU145 and LNCaP cells were incubated under humidified conditions at 37°C and 5% CO_2_. For passaging, the cancer cells were lifted by washing the flasks with HBSS buffer without calcium and magnesium (Invitrogen). After washing, the cells were incubated with trypsin and 0.05% EDTA (Invitrogen) for 2–5 min. Complete RPMI 1640 was added to the cells and they were collected into a 15 ml tube. The cells were then centrifuged at 300 ***g*** for 5 min. The supernatant was removed, and the cells were reseeded into flasks or used for experiments.

### Fluid shear stress treatment

The cancer cell lines were exposed to pulses of FSS using a protocol described previously (Barnes et al., 2012). The cancer cells were lifted as described above. The cells were collected into a 5 ml syringe (Thermo Fisher Scientific, Walter, MA, USA) at a density of 200,000 cells/ml. Poiseulle's equation was used to determine the maximum FSS exposure:(1)
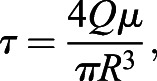
where *τ* is the wall shear stress in dyn/cm^2^, *Q* is the flow rate in cm^3^/s (14 ml/min), *μ* is the viscosity of the RPMI medium (assumed to be water at standard pressure and room temperature, or 0.01 dyn s cm^2^), and *R* is the inner radius of the 30 G needle (7.94×10^−3^ cm). The maximum FSS was computed to be 5920 dyn/cm^2^ (592 Pa). The local FSS exposure varies linearly with radial position, with the maximum FSS found at the wall of the conduit. Thus, the area-averaged FSS is equal to two-thirds of the maximum, or 3950 dyn/cm^2^ (395 Pa). The exposure time of a single pulse was 1.08 ms. The cancer cells were treated with 0, 1, 5 or 10 pulses of FSS. After FSS treatment, the cells were seeded into 24-well plates (CELLTREAT, Pepperell, MA, USA) at a density of 100,000 cells/well.

To justify the use of Poiseulle's equation for calculating the flow, the Reynolds number of the flow was calculated using the following equation:(2)
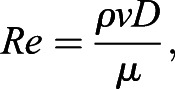
where *ρ* is the density of buffer, treated as water at standard pressure and room temperature (0.998 g/cm^3^), *v* is the velocity of flow, *D* is diameter of the inner needle and *μ* is the viscosity of the buffer. *Re* at a flow rate of 14 ml/min is 1850. This is below the threshold of 2200 for laminar flow, hence Poiseulle's equation is appropriate for the prediction of FSS.

### Annexin V/propidium iodide cell death assay

FITC-conjugated annexin V (AV; BD Pharmingen, San Diego, CA, USA) and propidium iodide (PI; BD Pharmingen) were used to assess cell viability 24 h after FSS treatment. The manufacturer's instructions were followed to prepare samples for flow cytometry. Viable cells were identified as being negative for both AV and PI, early apoptotic cells as being positive for AV only, necrotic cells as being positive for PI only and late apoptotic cells as being positive for both AV and PI.

Cells were incubated for 15 min with AV and PI at room temperature in the absence of light and immediately analyzed using a Guava easyCyte 12HT benchtop flow cytometer (MilliporeSigma, Burlington, MA, USA). Flow cytometry plots were analyzed using FlowJo software (FlowJo, Ashland, OR, USA). Flow cytometry plots of untreated static controls were used to draw gates (Fig. S1A). Cell death of cells pretreated with 20 µM CCD (Tocris Bioscience, Bristol, UK) or 50 µM Z-VAD-FMK (Tocris Bioscience) for 1 h prior to FSS exposure was also measured using this assay.

### Cell membrane damage and repair assay

For cell membrane damage, cells were incubated with PI for 5 min in RPMI 1640 medium. While still incubating with PI, the cells were loaded into a syringe and treated with FSS as described above. To measure repair, cancer cells were incubated with PI for 5 min at 1, 5, 10 and 20 min after FSS treatment. For both the damage and repair conditions, the cells were then washed with HBSS with calcium and magnesium and resuspended in HBSS with calcium and magnesium. The cells were analyzed via flow cytometry for PI fluorescence. Cells positive for PI were identified as cells with damaged membranes.

Cells were also stained with 10 µM concentrations of 3000, 10,000 and 40,000 MW FITC-tagged dextrans (Invitrogen) to assess cell membrane damage and repair. For membrane damage, cells were incubated with one of these dextrans in RPMI 1640 medium 5 min prior to FSS treatment. The cells were then treated with FSS, as described above, for 10 pulses. To measure membrane repair, the cells were incubated with the fluorescent dextrans 20 min after FSS treatment for 10 min. The cells were then washed with HBSS with calcium and magnesium and resuspended in HBSS with calcium and magnesium. Dextran fluorescence was measured using flow cytometry. Cells positive for dextran were identified as cells with damaged membranes. For both PI and dextran samples, drawing of cell damage gates was done using the static untreated control (Fig. S1B).

### Dextran–PI cell fate tracking

Cancer cells were incubated with 10 µM 3000 MW dextran 5 min prior to FSS. Cancer cells were treated with 10 pulses of FSS while still being incubated with the dextran. After FSS treatment, cancer cells were pelleted and washed with HBSS with calcium and magnesium to remove unabsorbed dextran. The cells were resuspended in complete RPMI 1640 medium and seeded onto a 24-well plate and incubated for 24 h at 37°C and 5% CO_2_. After incubating, the cells were lifted and stained with PI for 10 min in HBSS with calcium and magnesium. PI and dextran fluorescence were measured using a flow cytometer. Positive dextran fluorescence indicated cell membrane damage from FSS treatment, and PI fluorescence represented cell death. Static untreated controls were used to draw gates (Fig. S1C).

### Surface LAMP-1 staining

Cancer cells were treated with 10 or 0 pulses of FSS. 5 min after treatment, the cells were pelleted and stained with an Alexa Fluor 488-tagged antibody against surface LAMP-1 (Invitrogen, eBioH4A3, cat. #53-1079-42, lot #2134293) in 5% bovine serum albumin (Sigma-Aldrich, St Louis, MO, USA) at a 1:20 dilution. The cells were washed once with HBSS with calcium and magnesium and resuspended in HBSS with calcium and magnesium. Antibody staining was quantified using a flow cytometer. The untreated static controls were used to draw gates for surface LAMP-1 staining (Fig. S1D).

### Micropipette aspiration

Micropipette aspiration was performed as described in Taneja et al. (2020) to measure cell stiffness and relaxation time. Relaxation time was used as a measure of cell fluidity, with increased relaxation times being considered more fluid. To calculate stiffness the following equation was used:(3)
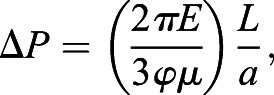
where Δ*P* is the change in suction pressure in Pa, *φ* is a constant term equal to 2.1, *μ* is the viscosity of the cytoplasm in Pa s, and *L*/*a* is normalized aspiration length. Relaxation time was calculated using the following equation:(4)
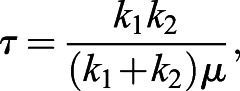
where *τ* is relaxation time in s, *μ* is the viscosity of the cytoplasm, and *k*_1_ and *k*_2_ are material parameters that can be solved via the following equation:(5)
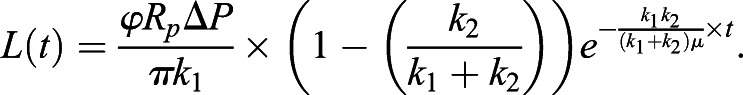


In Eqn. 5, *L*(*t*) is the aspiration length as a function time in units of µm and *t* is time in s. For micropipette aspiration measurements only, outliers were removed based on the following interquartile range criteria being true: *x*_*i*_>*x*_3_+(1.5×IQR) or *x*_*i*_<*x*_1_−(1.5×IQR), where *x*_*i*_ is an individual measurement, *x*_3_ is the third quartile value, *x*_1_ is the first quartile value and IQR is the interquartile range.

### *In vivo* shear stress study

Male eight-week-old nude (NU/NU) mice were purchased from the Jackson Laboratory (Bar Harbor, ME, USA). The study was approved by Institutional Review Board protocol #M1700009-00. All mice received identical care and housing conditions, and were monitored by veterinary staff in the Vanderbilt University Department of Animal Care (DAC).

One million DU145 and LNCaP cells were lifted per sample. The cells were counted using a hemocytometer. After resuspending the 1 million cells in 5 ml of medium, cells were treated with or without 10 pulses of FSS. The cells were not recounted after FSS treatment to account for cell death that was caused by the FSS treatment. This same *in vitro* shearing protocol was used extensively in this study, and representative viability data in parallel experiments are presented in the Results section. This cell death occurs when cancer cells pass through the circulatory system and is expected to have a significant impact on subsequent tumor growth. After the FSS treatment, the cells were resuspended in a 250 µl mixture of 1:1 phosphate-buffered saline and Matrigel (Corning Life Sciences, Tewksbury, MA, USA). The right flank was then injected with static DU145 cells and the left flank was injected with DU145 cells treated with 10 pulses of FSS. On another set of mice, the right flank was injected with static LNCaP cells and the left flank was injected with shear-treated LNCaP cells. Calipers were used to measure the length (L) and width (W) of each tumor. Tumor volume was estimated as (L×W^2^)/2. Mice in the study were euthanized at humane endpoints, as determined based on tumor size and recommendation by DAC veterinary staff.

### Statistical analysis

Data sets were plotted and analyzed using Prism 8 (GraphPad software, San Diego, CA, USA). A two-tailed unpaired *t*-test was used for statistical comparison between two groups, with *P*<0.05 considered significant. Least squares linear regression was used to determine whether slopes of fits significantly deviated from zero, with *P*<0.05 indicating statistical significance. Correlation was calculated using R^2^, which was calculated by simple linear regression. At least three independent replicates were used for each experiment.

## Supplementary Material

Supplementary information

Reviewer comments
