# Supplementary material for: Circulating prostate cancer cells have differential resistance to fluid shear stress-induced cell death
Source: J Cell Sci. 2021 Feb 22;134(4):jcs251470. doi: 10.1242/jcs.251470 (PMC7929932; doi:10.1242/jcs.251470)
Supplement: Supplementary information [file joces-134-251470-s1.pdf]

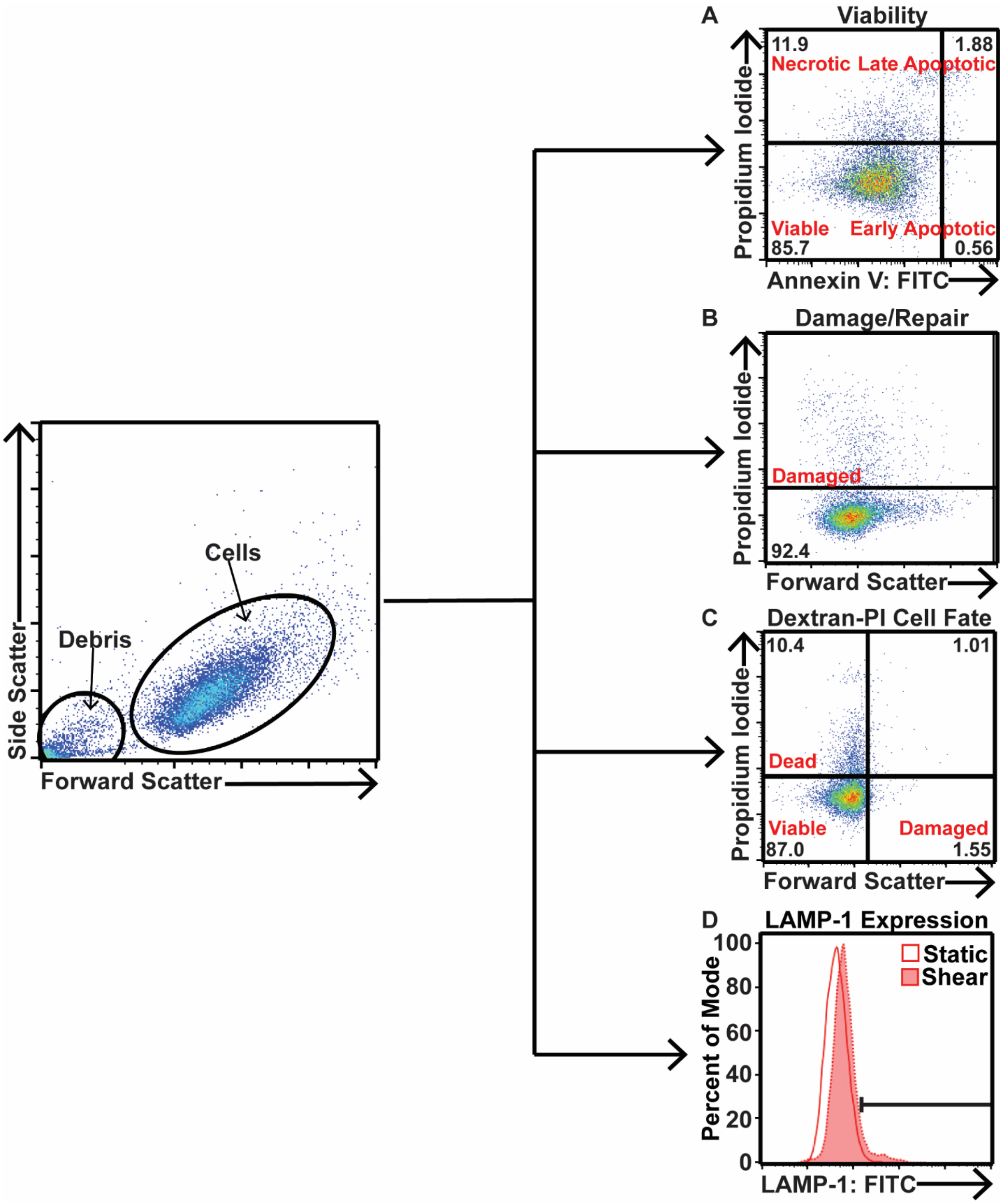

Figure S1 – Flow cytometry scatter plot gating examples. Forward and side scatter gating to isolate cells from debris. **A)** example flow cytometry gating of scatter and histogram plots of AV/PI stain, **B)** cell membrane damage/repair (same gating method was used for dextran cell membrane damage measurements), **C)** dextran-PI cell fate tracking, and **D)** LAMP-1 surface staining for DU145 static controls.

Journal of Cell Science • Supplementary information

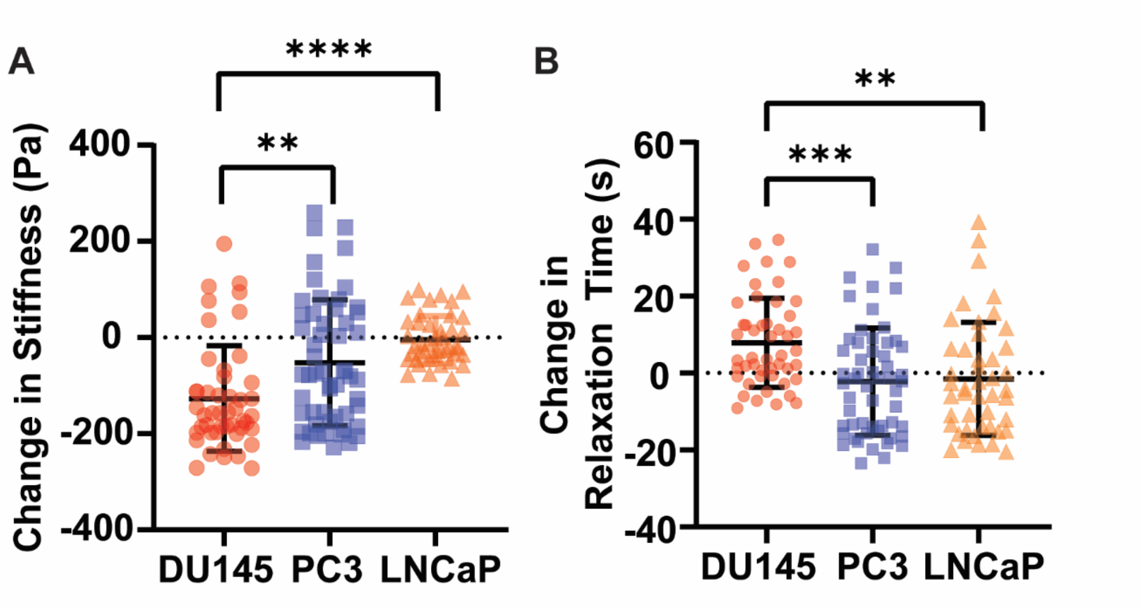

Figure S2 – change in biophysical properties of cancer cells after treatment with 10 pulses of FSS. A) Change in cancer cell stiffness in response to FSS treatment (DU145 – n =46; PC3 – n = 54; LNCaP – n = 41), and **B)** change in viscoelastic relaxation time following FSS exposure (DU145 – n = 48; PC3 – n = 54; LNCaP – n = 45). Mean and S.D. shown. N = 3 independent experiments of stiffness and relaxation time. ** $p < 0.01$, *** $p < 0.005$, **** $p < 0.001$. Unpaired two-tailed t test was used to compare groups.

Journal of Cell Science • Supplementary information
