# Supplementary material for: Circulating prostate cancer cells have differential resistance to fluid shear stress-induced cell death
Source: J Cell Sci. 2021 Feb 22;134(4):jcs251470. doi: 10.1242/jcs.251470 (PMC7929932; doi:10.1242/jcs.251470)
Supplement: Reviewer comments [file joces-134-251470_review_history.pdf]

Circulating prostate cancer cells have differential resistance to fluid shear stress-induced cell death

Jacob M. Hope, Matthew R. Bersi, Jenna A. Dombroski, Andrea B. Clinch, Rebecca S. Pereles, W. David Merryman and Michael R. King
DOI: 10.1242/jcs.251470

Editor: Andrew Ewald

Review timeline
Original submission: 9 July 2020
Editorial decision: 16 September 2020
First revision received: 4 November 2020
Editorial decision: 9 December 2020
Second revision received: 11 December 2020
Editorial decision: 11 January 2021
Third revision received: 12 January 2021
Accepted: 15 January 2021

Original submission

First decision letter

MS ID#: JOCES/2020/251470

MS TITLE: Death of circulating prostate cancer cells exposed to fluid shear stress correlates with increased cell membrane damage and cellular stiffness

AUTHORS: Jacob Hope, Matthew Bersi, Andrea Clinch, David Merryman, and Michael R. King
ARTICLE TYPE: Research Article

We have now reached a decision on the above manuscript.

To see the reviewers' reports and a copy of this decision letter, please go to: https://submit-jcs.biologists.org and click on the 'Manuscripts with Decisions' queue in the Author Area. (Corresponding author only has access to reviews.)

As you will see, the reviewers are enthusiastic about the subject of the study and many of its conclusions but raise a number of substantial criticisms that prevent me from accepting the paper at this stage. They suggest, however, that a revised version might prove acceptable, if you can address their concerns. If you think that you can deal satisfactorily with the criticisms on revision, I would be pleased to see a revised manuscript. We would then return it to the reviewers.

We are aware that you may currently be unable to access the lab to undertake experimental revisions. If it would be helpful, we encourage you to contact us to discuss your revision in greater detail. Please send us a point-by-point response indicating where you are able to address concerns raised (either experimentally or by changes to the text) and where you will not be able to do so within the normal timeframe of a revision. We will then provide further guidance. Please also note that we are happy to extend revision timeframes as necessary.

Please ensure that you clearly highlight all changes made in the revised manuscript. Please avoid using 'Tracked changes' in Word files as these are lost in PDF conversion.

© 2021. Published by The Company of Biologists under the terms of the Creative Commons Attribution License (https://creativecommons.org/licenses/by/4.0/).

I should be grateful if you would also provide a point-by-point response detailing how you have dealt with the points raised by the reviewers in the 'Response to Reviewers' box. Please attend to all of the reviewers' comments. If you do not agree with any of their criticisms or suggestions please explain clearly why this is so.

Reviewer 1

Advance summary and potential significance to field

The authors present and test a hypothesis for why different metastatic prostate cell lines have increased cell stiffness and reduced cell fluidity, i.e. because of their different primary origins and their dissemination through different vasculature and hemodynamics, the cells have a different 'force history.'

Comments for the author

Hope et. al. analyze the effects of high fluid shear stress on subsets of pancreatic cancer cells that have different patterns of metastatic propensity and primary tumor location. Notably, subsets of circulating tumor cells that travel through regions of arterial bifurcation or within the heart, such as DU145 prostate cancer cells, would experience FSSs greater than 1000 dyn/cm2, whereas circulating tumor cells that travel through the circulatory system or arterial system, such as PC3 or LNCaP prostate cancer cells, would be exposed to significantly lower FSSs. Accordingly, the authors hypothesized that the DU145 cells would exhibit resistance to high FSS, whereas the PC3 and LNCaP cells would show significantly decreased or lack of FSS resistance. To interrogate this hypothesis, the authors exposed the three cell lines to up to 10 pulses of 1.08 ms FSS of 3950 dyn/cm2 and quantified the extent of apoptosis, cell membrane damage and repair, and mitochondrial health. The results presented here indicate that FSS resistance of the interrogated prostate cancer cells lines likely correlates with increased cell stiffness and reduced cell fluidity. To solidify this conclusion, I believe that the following concerns should be addressed:

Major Concerns
1. The authors state that circulating cancer cells can experience FSS > 1000 dyn/cm^2 depending on circulation route, then state that they used a FSS of 3,950 dyn/cm^2 for a duration of 1.08 ms/pulse. However, they do not include a rationale for their chosen FSS and pulse duration. Additional justification is warranted.

2. In Figure 1A-C, the authors show the flow cytometry scatter plots of the three cell lines with gates drawn to divide the cells into four categories.
However, there is no inclusion of an explanation of how the gates were chosen. The authors should consider adding a supplemental figure with the scatter plots of the controls used for drawing the gates to justify the choice of the gate placement. This also applies to Figures 3A-C, 4A-C, and 5A-C.

3. The authors state “A previous study determined that PC3 prostate cancer cell lines were innately more resistant to FSS than healthy prostate epithelial cells, suggesting that FSS resistance contributes metastasis. This is consistent with the results of the present study where both DU145 and PC3 cells showed at least some resistance to FSS (Fig. 1).” However, the LNCaP cells do not appear to show resistance to FSS, and therefore, this claim is not supported by the results of Figure 1. To fully justify this claim, the authors should include an additional cell line, epithelial prostate cancer cells, and compare the results obtained using the three prostate cancer cells lines to the results obtained using epithelial prostate cancer cells or should edit the statement to accurately reflect their results.

4. The authors acknowledge that “… a dye of only one size was used in this study. This means that the precise size of the perforations in the cell membranes caused by FSS could not be determined.” However, this is important and more thoroughly interrogating the extent of membrane damage by quantifying the size of membrane perforations may provide a more complete rational for the differences in cell membrane repair and the extent of uncontrolled ion flux. Dyes of different diameters (such as a larger dextran) should be included in this study to better quantify perforation size.

© 2021. Published by The Company of Biologists under the terms of the Creative Commons Attribution License (https://creativecommons.org/licenses/by/4.0/).

5. The authors briefly state that Figure 6 suggest “… there may be an optimal cell stiffness for improved metastasis. A certain degree of cancer cell stiffness may allow a cancer cell to sufficiently navigate confined spaces and impart resistance to physical forces such as FSS.” This is a significant claim that is backed with only a few sentences. The authors should more thoroughly correlate the results of Figure 6 (including how changes in cell stiffness due to FSS exposure) with metastatic potential of the three cell lines.

6. Further, the authors state in the discussion that “… it was found that DU145 cells underwent a form of mechano-adaptation as they became softer and more fluid-like in response to FSS treatment (Fig. 6). As both LNCaP and PC3 cells were softer and experienced increased sensitivity to FSS, this mechano-adaptation likely does not promote FSS resistance.” This conclusion is unclear based on the brief explanation given, and, again, should be more thoroughly explained based on the figures presented in the paper.

7. There were some discrepancies with citation formatting pertaining to common inclusion (ex: … circulatory system11,12 versus >1,000 dyn/cm2 13) and grammatical errors (ex: Elevated FSS is known to cause cell membrane damage that can result in cell death to uncontrolled flux…) in the manuscript. The authors should readthrough and edit the paper prior to resubmission.

Minor Concerns
1. To improve the ease of interpreting the data, a consistent color scheme should be used for each cell line between bar graphs and line plots.

2. Given the substantial variation in static cell viability (Figure 1D), early-stage apoptosis (Figure 2A), necrosis (Figure 2C), and late-stage apoptosis (Figure 2E) among the different cell lines, plotting the accompanying line plots (Figures 1E, 2B,D,F) normalized to static conditions would improve the ease of comparing the effects of increasing pulses.

3. Similarly, normalization of Figures 3E, 4E, and 5E to static controls (FSS 0) for each cell line would improve interpretation of the results between cells lines instead of just between number of pulses or time.

4. Supplemental Figure 3 is a key figure for interpreting the results of Figure 4. Therefore, the authors should consider moving it to Figure 4.

5. Although not necessary, it would be interesting and informative to complete these studies at lower FSS’s (such as 1 dyn/cm^2 which would be experienced in venous compartments and 30 dyn/cm^2 which would be experienced in arterial circulation) and compare the results to those at very high FSS (> 1000 dyn/cm^2). This may inform if there is a threshold of FSS resistance of the different cell lines.

6. It is stated in the methods section that “Cells with depolarized mitochondria were identified as having low JC-1 red fluorescence and cells with healthy mitochondria were identified as having high red fluorescence. “. Quantification or descriptive flow cytometry plots of “low” and “high” fluorescence should be included.

7. Authors should quantitatively support their inferences about viability by using a statistical significance test to determine if the slope of each line is or is not 0. Similar comments are made regarding Figures 2B,D,F, 3E, 4E, 5E and should also include statistical analyses to justify claims regarding slopes.

Reviewer 2

Advance summary and potential significance to field

Hope and coworkers previously established an engineered high peak force shear stress model to test how hydrodynamic stress experienced by detached prostate cancer cells affects their viability, measured by flow cytometry several minutes up to 24 h later. In this work, they compared three prostate cancer cell lines and find clear differences of viability after 24 h, which were found

© 2021. Published by The Company of Biologists under the terms of the Creative Commons Attribution License (https://creativecommons.org/licenses/by/4.0/).

correlated with stress-induced mitochondrial depolarization and cell-intrinsic stiffness and relaxation time. They conclude that differences in the mechanical machinery cause cell-type specific differences of survival in circulation, and that the degree of cell membrane damage caused by shear stress correlates with enhanced cell death.

This work could be important in establishing cell-intrinsic stiffness or deformability as a key mechanism to resist shear stress in a fluid environment, with potential relevance for the ability of CTC to survive stress in circulation. However, in present form, technical and mechanistic weaknesses render this work as preliminary, descriptive, and therefore not conclusive.

Comments for the author

1. To reach sufficiently controlled technical and mechanistic level, the propidium iodide (PI) detection approach needs to be validated by an independent strategy. In Fig. 4, it is unexpected that the number of PI-
positive events does not change over 20 min, which may suggest that counterintuitively, membrane damage is not repaired in this setting. In addition, the fraction of early (Fig. 3, 4) and late (Fig. 1) PI positive events is not consistent, as numbers seem to increase with time, although no further mechanical challenge was imposed. This may suggest that certain damage types caused by shear stress are not reliably detected using the PI approach but still relevant for inducing cell death. The authors propose that some repair may be as rapid as within 1 min, without including any evidence for this idea. As a minimal technical validation of their approach, and to establish the chain of events, the authors should show membrane damage and repair directly, by detecting membrane repair complexes at very early and later time-points, ideally using microscopy. This could be readily achieved by fixing cells collected after imposing shear stress and performing antibody-
based detection.

2. It is unclear whether membrane damage detected by instantaneous PI uptake during shear stress (shown in Fig. 3) and cell death by necrosis and apoptosis later on are causally connected, as the authors suggest, or uncorrelated, e.g. due to repair. The authors should use a fate-tracing approach, e.g. dye uptake with a non-DNA binding reporter and measure the cells' commitment to survive or die. This would provide a causative link between the proposed damage event and the probability to survive or undergo necrosis / apoptosis.

3. In Fig. 5, JC-1 staining is used to report on "mitochondrial health".
Besides mitochondrial damage, JC-1 detection also identifies a switch from oxidative phosphorylation to glycolytic metabolism, which may be expected to occur in cancer cells as part of a physiologic stress response, and by EMT which can also be induced by shear stress (REF 56). Thus, LC-1 detection alone cannot discrimate between membrane damage-induced mitochondrial depolarization and decreased mitochondrial activity caused by other mechanisms. In addition the cell fraction showing low red signal are much smaller than PI positive subsets at the same time point (24 h), which is surprising considering that at least all PI positive cells (i.e., cells with damaged plasma membrane) would be expected to also show mitochondrial depolarization. Thus, in contrast to the authors' claims, it is unclear whether mitochondrial activity or damage are correlated or even causative for cell death. Positive controls for JC-1 transition, e.g. after pharmacological mitochondrial depolarization, followed by cell death measurements are required to show that the assay reliably reports the predicted "damage" function. Mitochondrial depolarization inhibitors, e.g. radical scavenger treatment, should be used to discriminate physiologic, non-fatal, from pathologic, fatal mitochondrial depolarization.
Lastly, can the authors show that the point of no return was reached in PI-positive cells? Therefore, dual-color detection of membrane damage and mitochondrial state need to be included for each condition.

4. The mechanistic link between cell stiffness and death events lack substantiation by a molecular intervention approach. In Fig. 6, the stiffness and relaxation time measurements were correlated with cell viability suggesting that stiffer cells survive shear stress better. However, other parameters than stiffness may be causative for cell viability, such as cell type dependent variability of damage repair or the state of cell cycle (which also affects cell stiffness) during the moment of stress. To demonstrate mechanistic relevance, cell stiffness should be modulated by molecular

© 2021. Published by The Company of Biologists under the terms of the Creative Commons Attribution License (https://creativecommons.org/licenses/by/4.0/).

intervention and the effect on cell viability show directly. As established approaches, Rho/ROCK inhibition or lamin downregulation should be considered.

Other points

5. The gating strategy to discriminate (pre)apoptotic from necrotic events (in Fig. 2) should be shown.
To support the claim of apoptosis directly and discriminate between necrotic from apoptotic events by an orthogonal approach, active caspase detection, or inhibition, should be included.

6. It is unclear how the data in Suppl. Fig. 3 and Fig. 4E are linked. Both datasets seem to show the change of PI over time, but only Suppl Fig. 3 shows recovery. This potential discordance needs to be clarified.

Reviewer 3

Advance summary and potential significance to field

In this research article, Hope et al. investigate the mechanisms of fluid shear stress (FSS) resistance displayed by some metastatic tumor cells. They use three human prostate cancer cell lines of three different metastatic origins with the rational that having had to survive drastically different range of FSS, these cell lines are differently equipped with means to survive FSS and will allow highlighting of the features that grant FSS resistance.

Structure review The introduction is solid, highlights why the questions raised are important and introduces the concepts that will come into play throughout the study well. Although the reasons behind the selection of the three cell lines are there, the rationale could be better articulated to make the relevance of comparing these three cell lines clearer. The choice of these cell lines (and the conclusions drawn) can also be questioned (see later comments).
In the first chapter of the results, the cell lines are exposed to FSS before cell viability is assessed. FSS treatment induces apoptosis in FSS-sensitive cells.
Next, the authors start investigating the reasons why apoptosis might be triggered by looking at potential membrane damage upon FSS treatment. A slightly unclear correlation was found between apoptosis and membrane damage, as the proportions of cells in apoptosis in DU145 and PC3 cell lines were different, but proportions of undamaged cells and ability to repair membrane damages seem very close between both cell lines.
The authors next look at mitochondrial health in their cell lines, as membrane damage causes uncontrolled ion flux that can induce mitochondrial dysfunction and apoptosis. The results are in line with the previous experiments, with DU145 cell line displaying higher mitochondrial health compared to PC-3, which explains why they display higher viability despite suffering comparable membrane damage.
The next section on cell stiffness lacks a proper introduction and one cannot intuitively understand why the authors switch from mitochondria health to cell stiffness. This could be improved if the authors spell out what they think the link between stiffness and apoptosis is (Is it that stiffness should play a role in the membraneÂ’s ability to resist FSS damage?) Micropipette aspiration is used to probe the cellÂ’s mechanical properties.
Results show that in this study, FSS-resistance seem to correlate with cell softening upon FSS exposure which is a bit puzzling and seemingly contrary to what the literature had previously been showing. The discussion logically goes through the obtained results and highlights the correlations that the results hinted at. However, the discussion needs to be toned down as issues (which are detailed either above or below) remain with the models used. The fact that their results clash with existing work in regard to cell stiffness changes upon FSS exposure also needs to be addressed and discussed here. I would thus recommend careful revision of this work to address the following issues.

Comments for the author

In this research article, Hope et al. investigate the mechanisms of fluid shear stress (FSS) resistance displayed by some metastatic tumor cells. They use three human prostate cancer cell lines of three different metastatic origins with the rational that having had to survive drastically different range

© 2021. Published by The Company of Biologists under the terms of the Creative Commons Attribution License (https://creativecommons.org/licenses/by/4.0/).

of FSS, these cell lines are differently equipped with means to survive FSS and will allow highlighting of the features that grant FSS resistance.

Structure review
The introduction is solid, highlights why the questions raised are important and introduces the concepts that will come into play throughout the study well. Although the reasons behind the selection of the three cell lines are there, the rationale could be better articulated to make the relevance of comparing these three cell lines clearer. The choice of these cell lines (and the conclusions drawn) can also be questioned (see later comments).
In the first chapter of the results, the cell lines are exposed to FSS before cell viability is assessed. FSS treatment induces apoptosis in FSS-sensitive cells.
Next, the authors start investigating the reasons why apoptosis might be triggered by looking at potential membrane damage upon FSS treatment. A slightly unclear correlation was found between apoptosis and membrane damage, as the proportions of cells in apoptosis in DU145 and PC3 cell lines were different, but proportions of undamaged cells and ability to repair membrane damages seem very close between both cell lines.
The authors next look at mitochondrial health in their cell lines, as membrane damage causes uncontrolled ion flux that can induce mitochondrial dysfunction and apoptosis. The results are in line with the previous experiments, with DU145 cell line displaying higher mitochondrial health compared to PC-3, which explains why they display higher viability despite suffering comparable membrane damage.
The next section on cell stiffness lacks a proper introduction and one cannot intuitively understand why the authors switch from mitochondria health to cell stiffness. This could be improved if the authors spell out what they think the link between stiffness and apoptosis is (Is it that stiffness should play a role in the membrane's ability to resist FSS damage?) Micropipette aspiration is used to probe the cell's mechanical properties.
Results show that in this study, FSS-resistance seem to correlate with cell softening upon FSS exposure which is a bit puzzling and seemingly contrary to what the literature had previously been showing.
The discussion logically goes through the obtained results and highlights the correlations that the results hinted at. However, the discussion needs to be toned down as issues (which are detailed either above or below) remain with the models used. The fact that their results clash with existing work in regard to cell stiffness changes upon FSS exposure also needs to be addressed and discussed here. I would thus recommend careful revision of this work to address the following issues.

General comments
While the subject is timely and the work is overall fairly logically structured, the results presented are mostly correlative and could be considered preliminary at this point.
The authors face the widespread issue of using cell lines that are subjected to cell culture bias and that often display different phenotypes already in static conditions, rendering the results obtained in FSS conditions less solid and less adapted to the drawing of conclusions on the mechanisms of FSS resistance in physiological conditions.
One step towards diminishing this issue could be to use tumor cells of identical metastatic origin but of different metastatic potential in parallel.
Their results obtained when measuring cell stiffness upon FSS exposure are surprising and opposite to what one could expect considering past published work on the subject.
Cell stiffening upon FSS exposure is now seemingly thought to be a part of the mechano-adaptive behavior that cells usually undergo upon FSS exposure (Moose et al. 2020 for example, FSS exposure = activation of myosin II, increase of cortical f-actin and thus increase of cell stiffness…)
Surprisingly in the study presented here the more a cell line is found to be FSS-resistant the softer it becomes upon FSS exposure (Fig 6).
More surprisingly, in "Alterations in cancer cell mechanical properties after fluid shear stress exposure: a micropipette aspiration study" – Chivukula, 2015, the same PC-3 cell line that the authors use in this study is found to undergo stiffening upon FSS exposure, using the same Micropipette Aspiration method for measuring mechanical properties.
The fact that the authors found opposite results when using the same cell line and the same method requires a comment. It is also likely yet another indicator that these cultured cell lines are not fully solid models due to cell culture biases. In addition, the authors could consider higher throughput techniques to probe viscoelastic behavior of cells to further consolidate their observations.

© 2021. Published by The Company of Biologists under the terms of the Creative Commons Attribution License (https://creativecommons.org/licenses/by/4.0/).

Specific points

1) Figure 1 would benefit from additional cell lines for increased relevance and demonstration that shear stress exposure decreasing cell viability can be very generally admitted and isn't at all specific to the prostate cancer cell lines used here.
2) Figure 1D: cell viability already seems a little different between the cell lines in static conditions. Are the cells differently sensitive to being put suspension? Differences in cell viability in FSS conditions might not entirely be due to the FSS treatment.
3) Figure 2 could be merged to figure 1 4) Figure 3: here again we have a case of cell lines showing very different phenotypes in static conditions already, with the LNCaP line showing significant membrane damage prior to FSS-treatment 5) Figure 4: same comment as figure 3 6) Figure 5: recuring issue of having cell lines display different numbers in static conditions already diminishing the importance of differences observed in the FSS conditions.
7) Figure 6: stiffness levels are already significantly different between cell lines in static conditions. Relative change in stiffness is thus displayed. Here, the trend is opposite to what could have been expected in light of existing work in the literature (see general comments)

First revision

Author response to reviewers' comments

We would like to first thank the reviewers for their comments and criticisms. We have incorporated their suggestions and believe that they have significantly improved the relevance and clarity of our study. We have also performed an in vivo *mouse study that improves the translational impact of the study. It is our hope that with these new experiments and clarifications the reviewers agree that this study is impactful and merits publication.*

Major Concerns

1. The authors state that circulating cancer cells can experience FSS > 1000 dyn/cm^2 depending on circulation route, then state that they used a FSS of 3,950 dyn/cm^2 for a duration of 1.08 ms/pulse. However, they do not include a rationale for their chosen FSS and pulse duration. Additional justification is warranted.

This oversight regarding the lack of explanation was corrected by adding an explanation regarding both the chosen FSS magnitude and the chosen pulse duration to the last paragraph in the Introduction section. It states: "This level of FSS was selected because a previous study identified this magnitude of FSS as representing a physiologically relevant level at which healthy-epithelial cells were sensitive to FSS for these brief pulses, whereas PC3 cancer cells were resistant (Barnes et al., 2012). Additionally, this elevated-FSS was studied in favor of lower FSS because previous studies have looked at the effect of lower FSS on CTC cell death (Huang et al., 2018; Lien et al., 2013; Mitchell and King, 2013b; Regmi et al., 2017). The short pulse duration was also selected because CTCs will primarily experience such elevated FSS for short durations on the order of ms (Strony et al., 1993)."

2. In Figure 1A-C, the authors show the flow cytometry scatter plots of the three cell lines with gates drawn to divide the cells into four categories. However, there is no inclusion of an explanation of how the gates were chosen. The authors should consider adding a supplemental figure with the scatter plots of the controls used for drawing the gates to justify the choice of the gate placement. This also applies to Figures 3A-C, 4A-C, and 5A-C.

The new Supplementary Figure 1 has been added to demonstrate how gating was performed for our flow cytometry results. The different gate regions were also labeled to indicate what each gate represents in terms of fluorescence. Sentences explaining how gates were selected using

© 2021. Published by The Company of Biologists under the terms of the Creative Commons Attribution License (https://creativecommons.org/licenses/by/4.0/).

untreated controls was added to each relevant flow cytometry method in the Methods section of the manuscript.

3. The authors state "A previous study determined that PC3 prostate cancer cell lines were innately more resistant to FSS than healthy prostate epithelial cells, suggesting that FSS resistance contributes metastasis. This is consistent with the results of the present study where both DU145 and PC3 cells showed at least some resistance to FSS (Fig. 1)." However, the LNCaP cells do not appear to show resistance to FSS, and therefore, this claim is not supported by the results of Figure 1. To fully justify this claim, the authors should include an additional cell line, epithelial prostate cancer cells, and compare the results obtained using the three prostate cancer cells lines to the results obtained using epithelial prostate cancer cells or should edit the statement to accurately reflect their results.

The quoted statement has been rewritten to eliminate the inconsistency and to more clearly explain that FSS resistance is not a conserved property across all prostate cancer cell lines. The statement in the Discussion section now states: "The results of this study showed that both DU145 and PC3 cells showed at least some resistance to FSS, whereas the LNCaP cells were quite sensitive to FSS (Figs. 1A-D)."

4. The authors acknowledge that "… a dye of only one size was used in this study. This means that the precise size of the perforations in the cell membranes caused by FSS could not be determined." However, this is important and more thoroughly interrogating the extent of membrane damage by quantifying the size of membrane perforations may provide a more complete rational for the differences in cell membrane repair and the extent of uncontrolled ion flux. Dyes of different diameters (such as a larger dextran) should be included in this study to better quantify perforation size.

Additional experiments were performed using dextrans of three different molecular weights: 3000 MW, 10,000 MW, and 40,000 MW to more precisely quantify the magnitudes of cell membrane damage during FSS or after membrane repair has been allowed to take place. These experiments are now described in the "FSS treatment causes differential cell membrane damage between prostate cancer cell lines" and "Cell membrane repair efficiency correlates with FSS resistance" portions of the results section. The experimental results are now summarized in Figures 2 and 3.

5. The authors briefly state that Figure 6 suggest "… there may be an optimal cell stiffness for improved metastasis. A certain degree of cancer cell stiffness may allow a cancer cell to sufficiently navigate confined spaces and impart resistance to physical forces such as FSS." This is a significant claim that is backed with only a few sentences. The authors should more thoroughly correlate the results of Figure 6 (including how changes in cell stiffness due to FSS exposure) with metastatic potential of the three cell lines.

Figure 6 has been changed to Figure 4. We carried out additional experiments in which the stiffness of DU145 and PC3 cells was reduced with cytochalasin D (CCD), a molecule that is known to reduce cell stiffness[1]. The results of these experiments involving CCD are summarized in Figure 4E and are discussed in both the results and discussion sections. For example, the fourth paragraph of the discussion section states: "This role of stiffness in FSS resistance was further supported by pretreating PC3 and DU145 cells with CCD, which is known to reduce cell stiffness (Rotsch and Radmacher, 2000). When PC3 and DU145 cells were treated with CCD, there was a significant reduction in normalized cell viability for cells also exposed to FSS (Fig. 4E)."

6. Further, the authors state in the discussion that "… it was found that DU145 cells underwent a form of mechano-adaptation as they became softer and more fluid-like in response to FSS treatment (Fig. 6). As both LNCaP and PC3 cells were softer and experienced increased sensitivity to FSS, this mechano-adaptation likely does not promote FSS resistance." This conclusion is unclear based on the brief explanation given, and, again, should be more thoroughly explained based on the figures presented in the paper.

This point has been better clarified using the results of the experiments involving CCD. The paper has been updated to state: "As treating PC3 and DU145 cells with CCD increased the cytotoxicity of FSS, this softening of DU145 likely does not promote FSS resistance (Fig. 4E)," meaning that

© 2021. Published by The Company of Biologists under the terms of the Creative Commons Attribution License (https://creativecommons.org/licenses/by/4.0/).

softened DU145 cells were not better at surviving FSS, so this change most likely does not help DU145 cells survive FSS. A hypothesis for how this change may affect DU145 metastasis is also offered stating: "Instead, the mechano-adaptation may promote the ability of DU145 cells to more efficiently extravasate and invade a secondary tumor site, since softer cells are associated with the ability to better navigate confined spaces (Wullkopf et al., 2018)."

7. There were some discrepancies with citation formatting pertaining to common inclusion (ex: … circulatory system11,12 versus >1,000 dyn/cm2 13) and grammatical errors (ex: Elevated FSS is known to cause cell membrane damage that can result in cell death to uncontrolled flux…) in the manuscript. The authors should readthrough and edit the paper prior to resubmission.

The authors have thoroughly edited the paper to correct these errors and others.

Minor Concerns

1. To improve the ease of interpreting the data, a consistent color scheme should be used for each cell line between bar graphs and line plots.

The color scheme has been revised to maintain consistency across all bar graphs and line plots.

2. Given the substantial variation in static cell viability (Figure 1D), early-stage apoptosis (Figure 2A), necrosis (Figure 2C), and late-stage apoptosis (Figure 2E) among the different cell lines, plotting the accompanying line plots (Figures 1E, 2B,D,F) normalized to static conditions would improve the ease of comparing the effects of increasing pulses.

The original Figure 2 has been combined with Figure 1. The normalized supplementary figures have been moved to the main figures. Subfigures that show the normalized percentage of cell death for early apoptosis, necrosis, and late death have been added to Figure 1. Additionally, the line plots of FSS pulses and different cell death states were normalized to static controls.

3. Similarly, normalization of Figures 3E, 4E, and 5E to static controls (FSS 0) for each cell line would improve interpretation of the results between cells lines instead of just between number of pulses or time.

The line plot normalizations stated in the previous response were carried out for these subfigures as well.

4. Supplemental Figure 3 is a key figure for interpreting the results of Figure 4. Therefore, the authors should consider moving it to Figure 4.

Supplementary Figure 3 has been moved to the main figure (now Figure 3).

5. Although not necessary, it would be interesting and informative to complete these studies at lower FSS's (such as 1 dyn/cm^2 which would be experienced in venous compartments and 30 dyn/cm^2 which would be experienced in arterial circulation) and compare the results to those at very high FSS (> 1000 dyn/cm^2). This may inform if there is a threshold of FSS resistance of the different cell lines.

Cell death induced by lower FSS over longer time periods have been studied in other papers[2-5]*. We feel that the novelty of this study is subjecting prostate cancer cells to elevated FSS that correlate with those found in the heart and in arterial bifurcations and in trying to understand why certain cells were more or less sensitive to these FSS. This is stated in the introduction: "Additionally, this elevated-FSS was studied in favor of lower FSS because previous studies have looked at the effect of lower FSS on CTC cell death (Huang et al., 2018; Lien et al., 2013; Mitchell and King, 2013b; Regmi et al., 2017)."*

6. It is stated in the methods section that "Cells with depolarized mitochondria were identified as having low JC-1 red fluorescence and cells with healthy mitochondria were identified as having high red fluorescence. ". Quantification or descriptive flow cytometry plots of "low" and "high" fluorescence should be included.

© 2021. Published by The Company of Biologists under the terms of the Creative Commons Attribution License (https://creativecommons.org/licenses/by/4.0/).

A scatter plot gating example for JC-1 red fluorescence has been included in the new Supplementary Figure 1E. An explanation of how the gates were selected was also added to the methods section for the JC-1 assay.

7. Authors should quantitatively support their inferences about viability by using a statistical significance test to determine if the slope of each line is or is not 0. Similar comments are made regarding Figures 2B,D,F, 3E, 4E, 5E and should also include statistical analyses to justify claims regarding slopes.

Least squares linear regression was performed for Figures 1D,H,I,J, 2D, 3D, 5B,D, and S2D (note the order of figures has changed). The results support the comments that were made in the initial submission and are now included in the paper to demonstrate whether the slope of each line significantly deviated from zero. The statistical tests are mentioned in the Methods section under statistical analysis and are included where relevant in figure captions.

Reviewer 2 Advance Summary and Potential Significance to Field:

Hope and coworkers previously established an engineered high peak force shear stress model to test how hydrodynamic stress experienced by detached prostate cancer cells affects their viability, measured by flow cytometry several minutes up to 24 h later. In this work, they compared three prostate cancer cell lines and find clear differences of viability after 24 h, which were found correlated with stress-induced mitochondrial depolarization and cell-intrinsic stiffness and relaxation time. They conclude that differences in the mechanical machinery cause cell-type specific differences of survival in circulation, and that the degree of cell membrane damage caused by shear stress correlates with enhanced cell death.

This work could be important in establishing cell-intrinsic stiffness or deformability as a key mechanism to resist shear stress in a fluid environment, with potential relevance for the ability of CTC to survive stress in circulation. However, in present form, technical and mechanistic weaknesses render this work as preliminary, descriptive, and therefore not conclusive.

Reviewer 2 Comments for the Author:
1. To reach sufficiently controlled technical and mechanistic level, the propidium iodide (PI) detection approach needs to be validated by an independent strategy. In Fig. 4, it is unexpected that the number of PI-positive events does not change over 20 min, which may suggest that, counterintuitively, membrane damage is not repaired in this setting. In addition, the fraction of early (Fig. 3, 4) and late (Fig. 1) PI positive events is not consistent, as numbers seem to increase with time, although no further mechanical challenge was imposed. This may suggest that certain damage types caused by shear stress are not reliably detected using the PI approach, but still relevant for inducing cell death. The authors propose that some repair may be as rapid as within 1 min, without including any evidence for this idea. As a minimal technical validation of their approach, and to establish the chain of events, the authors should show membrane damage and repair directly, by detecting membrane repair complexes at very early and later time-points, ideally using microscopy. This could be readily achieved by fixing cells collected after imposing shear stress and performing antibody-based detection.

An orthogonal strategy was used to detect membrane repair and damage. To determine the extent of cell membrane repair, LAMP-1 flow cytometry was used. LAMP-1 was used because it is known to translocate to the membrane surface for membrane repair[6]. The cells were not fixed prior to LAMP-1 staining because fixation has been shown to form small pores that can cause binding of the LAMP-1 antibody to internal LAMP-1[7]. The last paragraph in the "Repair is favored in DU145 cells" portion of the results section states: "DU145 and PC3 cells had a significant increase in surface LAMP-1 expression after FSS treatment, whereas LNCaP cells showed no change in LAMP-1 (Fig. 3I). This again indicates that membrane repair is not as efficient for LNCaP cells."
Additional experiments were performed using fluorescent dextrans of three different molecular weights: 3000 MW, 10,000 MW, and 40,000 MW. The presence of dextran within the cells was used to approximate the size of the cell membrane damage events. After 20 minutes was allowed for

© 2021. Published by The Company of Biologists under the terms of the Creative Commons Attribution License (https://creativecommons.org/licenses/by/4.0/).

cell membrane repair, "PC3 and LNCaP cells showed significantly greater uptake of 3,000 MW dextran, while DU145 cells did not (Fig. 3E)."
The statement about repair taking place within 1 minute was removed. Although staining was not done to look more specifically at the rate of repair, we feel that these two other methods of assessing cell membrane repair better support our statement that cell membrane repair was occurring following FSS treatment.
Together, the results of these assays showed that cell membrane repair efficiency "correlated with how well a cancer cell line resisted FSS-induced cell death with LNCaP cells being the most sensitive and the least efficient at membrane repair, while DU145 cells had the most efficient repair and experienced the least amount of cell death."

2. It is unclear whether membrane damage detected by instantaneous PI uptake during shear stress (shown in Fig. 3) and cell death by necrosis and apoptosis later on are causally connected, as the authors suggest, or uncorrelated, e.g. due to repair. The authors should use a fate-tracing approach, e.g. dye uptake with a non-DNA binding reporter and measure the cells' commitment to survive or die. This would provide a causative link between the proposed damage event and the probability to survive or undergo necrosis / apoptosis.

The dextran/PI experiments were used as a fate-tracing approach, where cells were incubated with 3000 MW dextran during and prior to FSS exposure to measure damage. The cells were then stained with PI the next day to measure cell death. These results are shown in Figures 2G,H. The results indicate that cells with dextran uptake were more likely to take up PI stain (indicating death) than those that did not. The link between the damage events and the probability of survival has been further explained in the "FSS treatment causes differential cell membrane damage between prostate cancer cell lines" portion of the results section. A detailed description was given: "For all three cell lines, the proportion of PI-positive cells was significantly increased for dextran-positive cells. This indicates that cells damaged by FSS are more likely to undergo cell death by FSS treatment compared to undamaged cells. LNCaP cells that were damaged had a significantly greater proportion of PI-positive cells than PC3 and DU145 cells, again indicating that LNCaP cells are more sensitive to FSS-induced cell death (Fig. 2H)."

3. In Fig. 5, JC-1 staining is used to report on "mitochondrial health". Besides mitochondrial damage, JC-1 detection also identifies a switch from oxidative phosphorylation to glycolytic metabolism, which may be expected to occur in cancer cells as part of a physiologic stress response, and by EMT which can also be induced by shear stress (REF 56). Thus, LC-1 detection alone cannot discrimate between membrane damage-induced mitochondrial depolarization and decreased mitochondrial activity caused by other mechanisms. In addition, the cell fraction showing low red signal are much smaller than PI positive subsets at the same time point (24 h), which is surprising considering that at least all PI positive cells (i.e., cells with damaged plasma membrane) would be expected to also show mitochondrial depolarization. Thus, in contrast to the authors' claims, it is unclear whether mitochondrial activity or damage are correlated or even causative for cell death. Positive controls for JC-1 transition, e.g. after pharmacological mitochondrial depolarization, followed by cell death measurements are required to show that the assay reliably reports the predicted "damage" function. Mitochondrial depolarization inhibitors, e.g. radical scavenger treatment, should be used to discriminate physiologic, non-fatal, from pathologic, fatal mitochondrial depolarization. Lastly, can the authors show that the point of no return was reached in PI-positive cells? Therefore, dual-color detection of membrane damage and mitochondrial state need to be included for each condition.

We thank the reviewer for this suggestion to investigate the mitochondrial dysfunction data further. Since there was no major change found in mitochondrial health for cells treated with FSS, these data were considered less central to the manuscript overall. The corresponding figure (Figure 5) has been moved to Supplementary Figure 2. Instead we prioritized other experiments that we believed would be more impactful, such as the newly added mouse experiment. To reflect this change, the "FSS treatment reduces mitochondrial health" portion of the results section has been removed. Instead, a brief description of the JC-1 flow cytometry experiments is given in the last paragraph of the "FSS treatment induces cell death in PC3 and LNCaP cells" portion of the results section.

© 2021. Published by The Company of Biologists under the terms of the Creative Commons Attribution License (https://creativecommons.org/licenses/by/4.0/).

4. The mechanistic link between cell stiffness and death events lack substantiation by a molecular intervention approach. In Fig. 6, the stiffness and relaxation time measurements were correlated with cell viability, suggesting that stiffer cells survive shear stress better. However, other parameters than stiffness may be causative for cell viability, such as cell type dependent variability of damage repair or the state of cell cycle (which also affects cell stiffness) during the moment of stress. To demonstrate mechanistic relevance, cell stiffness should be modulated by molecular intervention and the effect on cell viability show directly. As established approaches, Rho/ROCK inhibition or lamin downregulation should be considered.

To demonstrate mechanistic relevance, cytochalasin D (CCD) was used to reduce the stiffness of DU145 and PC3 cells. CCD has previously been shown to reduce cell stiffness[1]. The less stiff cells showed reduced normalized cell viability to FSS. These results are highlighted in Figure 4E. A discussion of this has been added to the fourth paragraph of the discussion section, stating: "When PC3 and DU145 cells were treated with CCD, there was a significant reduction in normalized cell viability for cells also exposed to FSS (Fig. 4E). Likewise, a previous study that reduced lamin A/C expression, a protein known to promote cell stiffness, also had the effect of sensitizing cancer cells to FSS (Lee et al., 2007; Mitchell et al., 2015)."

Other points

5. The gating strategy to discriminate (pre)apoptotic from necrotic events (in Fig. 2) should be shown. To support the claim of apoptosis directly and discriminate between necrotic from apoptotic events by an orthogonal approach, active caspase detection, or inhibition, should be included.
The new Supplementary Figure 1 has been created to show the gating strategy used to discriminate apoptotic from necrotic events. The gate labels are also shown in Figure 1A. The pan-caspase inhibitor Z-VAD-FMK was used to determine if cell death in LNCaP cells was apoptotic or necrotic in nature. Figure 1K was added to summarize the results of this experiment, and a brief description is now included in the "FSS treatment induces cell death in PC3 and LNCaP cells" portion of the results section. For example, the last sentence of the third paragraph in that portion states: "Z-VAD-FMK did not significantly increase cell viability in response to FSS treatment, suggesting that the cell death was necrotic rather than apoptotic (Fig. 1K)."

6. It is unclear how the data in Suppl. Fig. 3 and Fig. 4E are linked. Both datasets seem to show the change of PI over time, but only Suppl Fig. 3 shows recovery. This potential discordance needs to be clarified.

Supplementary Figure 3 has been moved to Figure 3C. Figure 4E has been moved to Figure 3D. The difference between the two figures is that 3C refers to cells damaged during FSS, such as in Figure 2, and cells that have been stained with PI 20 min after FSS treatment to allow membrane repair to take place. The purpose of this subfigure is to assess if there is a change in the damaged populations from the time during FSS to 20 min after FSS. 4D is meant to show if there was any change in the damaged population from 1 to 20 min after FSS treatment. In other words, are fewer cells damaged at 20 min post-FSS compared to 1 min post-FSS? The results indicate that this is not the case. These points are made in the results section by stating: "The undamaged cell populations after 20 min of FSS exposure for each cell line were normalized to their untreated controls. When these normalized repair populations were compared to the normalized population of cells damaged during FSS treatment, there was a significant increase in the normalized undamaged cell populations for the repair condition in each cell line (Fig. 3C). Taken together, these results suggest that while some of the cancer cells from each cell line are permanently damaged by FSS, repair does take place for many of the damaged cells. When comparing the 20 min repair conditions of each cell line, DU145 and PC3 cells had significantly higher normalized undamaged cell populations than the LNCaP cells, indicating that LNCaP cells suffered more irreversible damage by FSS (Fig. 3C). To assess if each cell line had a different rate of repair, cell membrane damage was measured from 1 to 20 min following 10 pulses of FSS treatment. For each cell line, there was no major change in the undamaged cell population, even when comparing the 1 and 20 min groups (Fig. 3D)." These differences are also highlighted in the Figure 3 caption by stating: "C) Average proportion of undamaged cells for cell membrane damage measurements taken during FSS (damage) and for cells given 20 min of repair time after FSS (repair). D)

© 2021. Published by The Company of Biologists under the terms of the Creative Commons Attribution License (https://creativecommons.org/licenses/by/4.0/).

Normalized PI-negative undamaged cancer cells after different cell membrane repair times, ranging from 1 to 20 min."

Reviewer 3 Advance Summary and Potential Significance to Field:
In this research article, Hope et al. investigate the mechanisms of fluid shear stress (FSS) resistance displayed by some metastatic tumor cells. They use three human prostate cancer cell lines of three different metastatic origins with the rational that having had to survive drastically different range of FSS, these cell lines are differently equipped with means to survive FSS and will allow highlighting of the features that grant FSS resistance.

Structure review
The introduction is solid, highlights why the questions raised are important and introduces the concepts that will come into play throughout the study well. Although the reasons behind the selection of the three cell lines are there, the rationale could be better articulated to make the relevance of comparing these three cell lines clearer. The choice of these cell lines (and the conclusions drawn) can also be questioned (see later comments).
In the first chapter of the results, the cell lines are exposed to FSS before cell viability is assessed. FSS treatment induces apoptosis in FSS-sensitive cells.
Next, the authors start investigating the reasons why apoptosis might be triggered by looking at potential membrane damage upon FSS treatment. A slightly unclear correlation was found between apoptosis and membrane damage, as the proportions of cells in apoptosis in DU145 and PC3 cell lines were different, but proportions of undamaged cells and ability to repair membrane damages seem very close between both cell lines.
The authors next look at mitochondrial health in their cell lines, as membrane damage causes uncontrolled ion flux that can induce mitochondrial dysfunction and apoptosis. The results are in line with the previous experiments, with DU145 cell line displaying higher mitochondrial health compared to PC-3, which explains why they display higher viability despite suffering comparable membrane damage. The next section on cell stiffness lacks a proper introduction and one cannot intuitively understand why the authors switch from mitochondria health to cell stiffness. This could be improved if the authors spell out what they think the link between stiffness and apoptosis is (Is it that stiffness should play a role in the membrane's ability to resist FSS damage?) Micropipette aspiration is used to probe the cell's mechanical properties. Results show that in this study, FSS-resistance seem to correlate with cell softening upon FSS exposure, which is a bit puzzling and seemingly contrary to what the literature had previously been showing. The discussion logically goes through the obtained results and highlights the correlations that the results hinted at. However, the discussion needs to be toned down as issues (which are detailed either above or below) remain with the models used. The fact that their results clash with existing work in regard to cell stiffness changes upon FSS exposure also needs to be addressed and discussed here. I would thus recommend careful revision of this work to address the following issues.

General comments
While the subject is timely and the work is overall fairly logically structured, the results presented are mostly correlative and could be considered preliminary at this point.
The authors face the widespread issue of using cell lines that are subjected to cell culture bias and that often display different phenotypes already in static conditions, rendering the results obtained in FSS conditions less solid and less adapted to the drawing of conclusions on the mechanisms of FSS resistance in physiological conditions.
One step towards diminishing this issue could be to use tumor cells of identical metastatic origin but of different metastatic potential in parallel.
Their results obtained when measuring cell stiffness upon FSS exposure are surprising and opposite to what one could expect considering past published work on the subject.
Cell stiffening upon FSS exposure is now seemingly thought to be a part of the mechano-adaptive behavior that cells usually undergo upon FSS exposure (Moose et al. 2020 for example, FSS exposure = activation of myosin II, increase of cortical f-actin and thus increase of cell stiffness…)
Surprisingly in the study presented here the more a cell line is found to be FSS-resistant the softer it becomes upon FSS exposure (Fig 6).
More surprisingly, in "Alterations in cancer cell mechanical properties after fluid shear stress exposure: a micropipette aspiration study" – Chivukula, 2015, the same PC-3 cell line that the

© 2021. Published by The Company of Biologists under the terms of the Creative Commons Attribution License (https://creativecommons.org/licenses/by/4.0/).

authors use in this study is found to undergo stiffening upon FSS exposure, using the same Micropipette Aspiration method for measuring mechanical properties.
The fact that the authors found opposite results when using the same cell line and the same method requires a comment. It is also likely yet another indicator that these cultured cell lines are not fully solid models due to cell culture biases. In addition, the authors could consider higher throughput techniques to probe viscoelastic behavior of cells to further consolidate their observations.

Specific points

1) Figure 1 would benefit from additional cell lines for increased relevance and demonstration that shear stress exposure decreasing cell viability can be very generally admitted and isn't at all specific to the prostate cancer cell lines used here.

We thank the reviewer for this comment to investigate additional cell lines. This paper was intended to focus solely on prostate cancer cells. Other projects in the lab are looking at other various cancer cell types. In particular, there is ongoing work to determine if chemoresistant cells are less sensitive to FSS-induced cell death.

2) Figure 1D: cell viability already seems a little different between the cell lines in static conditions. Are the cells differently sensitive to being put suspension? Differences in cell viability in FSS conditions might not entirely be due to the FSS treatment.

To account for variations in cell phenotypes, normalized data was included in Figure 1H-J. However, we do agree with the reviewer that these possibly different sensitivities of these cells to being in suspension is a potential limitation of this study, as well as other discrepancies in the cell phenotypes that cannot be controlled for in using these 3 cell lines. This limitation is now mentioned in the discussion: "It is important to note that these cancer cell lines were derived from different patients. Thus, there is significant genetic heterogeneity among these cell lines. To more conclusively identify if properties such as stiffness promote FSS resistance, it would be interesting to determine if cancer cell lines of different metastatic potentials that are derived from the same patient have similar results to those presented in this study." To address this limitation, the lab is currently developing a shear resistant line of LNCaP cells from the FSS-sensitive LNCaP cells. This work, in our opinion, merits a thorough study that would be best presented in a future manuscript. We believe this manuscript provides a good foundation for future projects to better determine what phenotypes promote FSS resistance.

3) Figure 2 could be merged to figure 1

Figure 2 is now merged with Figure 1.

4) Figure 3: here again we have a case of cell lines showing very different phenotypes in static conditions already, with the LNCaP line showing significant membrane damage prior to FSS-treatment

Normalizations were done for comparison. See response for comment 2 about addressing this limitation in the discussion.

5) Figure 4: same comment as figure 3

Normalizations were done for comparison. See response for comment 2 about addressing this limitation in the discussion.

6) Figure 5: recuring issue of having cell lines display different numbers in static conditions already, diminishing the importance of differences observed in the FSS conditions.

Normalized data has been included in these figures to help account for variations in cell phenotypes. An acknowledgement regarding genetic diversity in the cells used for this study has been added to the discussion section. Future work is suggested as well with the statement: "It is important to note that these cancer cell lines were derived from different patients. Thus, there is

© 2021. Published by The Company of Biologists under the terms of the Creative Commons Attribution License (https://creativecommons.org/licenses/by/4.0/).

significant genetic heterogeneity among these cell lines. To more conclusively identify if properties such as stiffness promote FSS resistance, it would be interesting to determine if cancer cell lines of different metastatic potentials that are derived from the same patient have similar results to those presented in this study."

7) Figure 6: stiffness levels are already significantly different between cell lines in static conditions. Relative change in stiffness is thus displayed. Here, the trend is opposite to what could have been expected in light of existing work in the literature (see general comments)

Cell membrane damage can induce a decrease in cell stiffness by disassembling the cytoskeleton. This disassembly reduces the cell membrane tension that resists hydrophobic forces, which cause cell membranes to heal[8]. A comment regarding the discrepancy has been added to the discussion section, stating: "The reduced stiffness of FSS-treated DU145 cells was unexpected as a previous study that used the same method of FSS treatment found that PC3 cells became stiffer after FSS treatment (Chivukula et al., 2015). However, FSS exposure has also previously been shown to reduce cell stiffness (Xin et al., 2019). One possibility for this discrepancy is the divergence of cells cultured in different labs for different lengths of time. Another explanation for the results of this study might be that the stiffness was immediately analyzed following FSS exposure. Cell membrane repair traditionally requires cytoskeleton disassembly to relieve tension in the cell, which reduces the force needed for membrane wound closure (Abreu-Blanco et al., 2012). This cytoskeletal disassembly causes cell softening (Kasas et al., 2005). However, at later time points in membrane repair, these cytoskeleton proteins are replaced, possibly causing the increased stiffness observed in the previous study (Abreu-Blanco et al., 2012; Chivukula et al., 2015)."

References:
1. Rotsch, C. & Radmacher, M. Drug-Induced Changes of Cytoskeletal Structure and Mechanics in Fibroblasts: An Atomic Force Microscopy Study. *Biophys. J.* **78**, 520–535 (2000).
2. Huang, Q. *et al.* Fluid shear stress and tumor metastasis. *Am. J. Cancer Res.* **8**, 763–777 (2018).
3. Lien, S.-C. *et al.* Mechanical regulation of cancer cell apoptosis and autophagy: Roles of bone morphogenetic protein receptor, Smad1/5, and p38 MAPK. *Biochim. Biophys. Acta BBA - Mol. Cell Res.* **1833**, 3124–3133 (2013).
4. Regmi, S., Fu, A. & Luo, K. Q. High Shear Stresses under Exercise Condition Destroy Circulating Tumor Cells in a Microfluidic System. *Sci. Rep.* **7**, 39975 (2017).
5. Mitchell, M. J. & King, M. R. Fluid shear stress sensitizes cancer cells to receptor-mediated apoptosis via trimeric death receptors. *New J. Phys.* **15**, 015008 (2013).
6. Reddy, A., Caler, E. V. & Andrews, N. W. Plasma Membrane Repair Is Mediated by Ca2+-Regulated Exocytosis of Lysosomes. *Cell* **106**, 157–169 (2001).
7. Corrotte, M., Castro-Gomes, T., Koushik, A. B. & Andrews, N. W. Approaches for plasma membrane wounding and assessment of lysosome-mediated repair responses. *Methods Cell Biol.* **126**, 139–158 (2015).
8. Abreu-Blanco, M. T., Watts, J. J., Verboon, J. M. & Parkhurst, S. M. Cytoskeleton Responses in Wound Repair. *Cell. Mol. Life Sci.* **69**, 2469–2483 (2012).

Second decision letter

MS ID#: JOCES/2020/251470

MS TITLE: Circulating prostate cancer cells have differential resistance to fluid shear stress-induced cell death

AUTHORS: Jacob Hope, Matthew R. Bersi, Jenna A. Dombroski, Andrea Clinch, Rebecca S. Pereles, David Merryman, and Michael R. King
ARTICLE TYPE: Research Article

We have now reached a decision on the above manuscript.

© 2021. Published by The Company of Biologists under the terms of the Creative Commons Attribution License (https://creativecommons.org/licenses/by/4.0/).

To see the reviewers' reports and a copy of this decision letter, please go to: https://submit-jcs.biologists.org and click on the 'Manuscripts with Decisions' queue in the Author Area. (Corresponding author only has access to reviews.)

As you will see, the reviewers agree that a number of issues raised in the first round of review have been successfully resolved. However, reviewer 2 correctly identifies that the crucial in vivo experiments are not fully satisfactory. Subcutaneous implantation is not typically used as a model system for metastasis- more commonly it is for primary tumor growth. Since the focus of the study is on the impact of fluid shear stress on distant organ colonization it is unclear why intravenous injection was not used, with readouts at the level of metastasis to lung or bones. These experiments can be quite rapid and definitive in testing conclusions like yours. Reviewer 2 suggests, however, that a revised version might prove acceptable, if you can address their concerns. If you think that you can deal satisfactorily with the criticisms on revision, I would be pleased to see a revised manuscript. We would then return it to reviewer 2 specifically. Please let me know if you have questions.

We are aware that you may be experiencing disruption to the normal running of your lab that makes experimental revisions challenging. If it would be helpful, we encourage you to contact us to discuss your revision in greater detail. Please send us a point-by-point response indicating where you are able to address concerns raised (either experimentally or by changes to the text) and where you will not be able to do so within the normal timeframe of a revision. We will then provide further guidance. Please also note that we are happy to extend revision timeframes as necessary.

Please ensure that you clearly highlight all changes made in the revised manuscript. Please avoid using 'Tracked changes' in Word files as these are lost in PDF conversion.

I should be grateful if you would also provide a point-by-point response detailing how you have dealt with the points raised by the reviewers in the 'Response to Reviewers' box. Please attend to all of the reviewers' comments. If you do not agree with any of their criticisms or suggestions please explain clearly why this is so.

<u>Reviewer 1</u>

Advance summary and potential significance to field

The authors present and test a hypothesis for why different metastatic prostate cell lines have increased cell stiffness and reduced cell fluidity, i.e. because of their different primary origins and their dissemination through different vasculature and hemodynamics, the cells have a different 'force history.'

Comments for the author

The authors sufficiently edited the paper to address my initial concerns. The most valuable addition was the in vivo experimental study of tumor growth in mice injected with cells exposed to FSS vs. cells not exposed to shear stress. They also addressed the other comments I had regarding gating explanations, the addition of different size dextran particles to measure cell membrane damage, and better statistical analysis to support their conclusions. I do not have further concerns.

<u>Reviewer 2</u>

Advance summary and potential significance to field

See previous review

Comments for the author

The authors have included new experiments on fate-tracing using dextran uptake, which demonstrate that a 60% subset of dying cells are indeed dextran-positive. This indicates that an

© 2021. Published by The Company of Biologists under the terms of the Creative Commons Attribution License (https://creativecommons.org/licenses/by/4.0/).

early membrane damage is associated with cell death. Additional data show the gating strategy, and additional data supporting necrotic, rather than apoptotic cell death after shear stress.

As a major new shortcoming, the authors included new preliminary and not fully conclusive mouse data (new Fig. 5). Cells were subjected to shear stress implanted subcutaneously, and then monitored for growth over 4 weeks. First the growth in the mouse skin of prostate cancer cells cannot be considered orthotopic, hence the relevance for this work is unclear. Second, the relevance of the administration route is questionable, since tumor cells experiencing shear stress during passage in the blood would be expected to colonize distant organs. This can be modelled by trail vein injection and monitoring of lung and, in prostate cancer, bone metastasis, but not subcutaneous injection. The approach is poorly described. How many cells were implanted? How was an equal number of viable cells guaranteed, given differences of PI positivity after the shear stress experiment between cell lines? In addition, data on PC3 cells in this setting are lacking. If the authors wish to demonstrate differential long-term viability/"fitness" of the cells after shear stress, hematogenous administration should be performed.

Alternatively, differential colony-forming capacity in an anchorage-independent assay could be considered. As presented here, the data in Fig. 5 harm, rather than support this otherwise interesting work.

The section "FSS treatment reduces mitochondrial health" was removed, however the data on JC-1 staining were retained (Suppl. Fig. 2). Due to the puzzling finding that the stress response and membrane damage (in 50% of the cells) barely associates with mitochondrial depolarization, the data remain inconclusive and even conflicting, and fail to support the overall direction.
The authors should consider removing this part or adding meaningful controls to consolidate the main claim, such as those proposed during the first review round.

Cytochalasin treatment moderately reduced viability of PC3 and DU145 cells after shear stress, which akes sense, albeit the effect size is surprisingly small. The authors should still add the data for LNCap cells, to demonstrate consistency.

Minor
p. 5 top: "24 h FSS treatment cell death" should read: 24 h after FSS treatment cell death

Reviewer 3

Advance summary and potential significance to field

In this research article, Hope et al. investigate the mechanisms of fluid shear stress (FSS) resistance displayed by some metastatic tumor cells. They use three human prostate cancer cell lines of three different metastatic origins with the rational that having had to survive drastically different range of FSS, these cell lines are differently equipped with means to survive FSS and will allow highlighting of the features that grant FSS resistance.

Comments for the author

The authors have now addressed carefully most of the issues raised in the first round.

Second revision

Author response to reviewers' comments

We thank the reviewers for their comments and criticisms. We have addressed their comments with revisions to the text and believe that these edits have improved the clarity of the paper and better support the stated conclusions.

© 2021. Published by The Company of Biologists under the terms of the Creative Commons Attribution License (https://creativecommons.org/licenses/by/4.0/).

Reviewer 2 Comments for the Author:
The authors have included new experiments on fate-tracing using dextran uptake, which demonstrate that a 60% subset of dying cells are indeed dextran-positive. This indicates that an early membrane damage is associated with cell death. Additional data show the gating strategy, and additional data supporting necrotic, rather than apoptotic cell death after shear stress.

As a major new shortcoming, the authors included new preliminary and not fully conclusive mouse data (new Fig. 5). Cells were subjected to shear stress, implanted subcutaneously, and then monitored for growth over 4 weeks. First, the growth in the mouse skin of prostate cancer cells cannot be considered orthotopic, hence the relevance for this work is unclear. Second, the relevance of the administration route is questionable, since tumor cells experiencing shear stress during passage in the blood would be expected to colonize distant organs. This can be modelled by trail vein injection and monitoring of lung and, in prostate cancer, bone metastasis, but not subcutaneous injection. The approach is poorly described. How many cells were implanted? How was an equal number of viable cells guaranteed, given differences of PI positivity after the shear stress experiment between cell lines? In addition, data on PC3 cells in this setting are lacking. If the authors wish to demonstrate differential long-term viability/"fitness" of the cells after shear stress, hematogenous administration should be performed. Alternatively, differential colony-forming capacity in an anchorage-independent assay could be considered. As presented here, the data in Fig. 5 harm, rather than support this otherwise interesting work.

The issues that Reviewer 2 addresses in this comment are important for consideration, and deserve discussion here and in the manuscript. We note that Reviewer 1 feels that the mouse data added significant impact to the study. We believe that the experimental design originally used in the manuscript and presented in Figure 5 is appropriate for answering the questions that we asked in this study, but this may not have been clear from our original presentation.

Orthotopic and injection assays by tail vein or intracardiac injection are traditional methods of assaying metastasis in mice. Our lab has previously employed these methods in other studies to determine treatment efficacy to prevent metastasis, or in assaying how different proteins affect metastatic colonization (Li et al., 2013; Mitchell et al., 2014). Here, we have elected to isolate the effect of shear pre-conditioning, without the other confounding factors involved from intravenous circulation to extravasation and engraftment. The value of the subcutaneous method used is that we have control over the extent and magnitude of FSS exposure of these cell lines. When injecting the cancer cells into mice intravenously (IV), they will then be exposed to a variety of other FSS that we cannot control or specifically identify. We believe it is also likely that the effect of pretreating the LNCaP cells with FSS would be voided by the constant and variable FSS that they would experience after IV injection. A previous study showed that untreated LNCaP cells were unable to form metastatic lesions after tail vein or intracardiac injection (Wu et al., 1998). This effect would likely carry over to an orthotopic model, where the LNCaP cells would experience similar FSS to IV injection eventually as they attempt to disseminate to other sites. Mutations may occur over the time course of the experiment in the LNCaP cells that allow for secondary sites to be colonized, but this would not be relevant to this study as the LNCaP cells would presumably have different properties from those LNCaP cells originally assayed. Those potentially different properties that allow for LNCaP distant metastasis would be enlightening as future work. However, we addressed this idea in the last round of revisions in stating our goal of developing a shear resistant LNCaP line to compare to the parental line for a different, future study. This motivation was not sufficiently explained in our last submission.

There are other confounding factors in orthotopic or injection assays that would obscure the isolated effect of controlled shear exposure, including differences in size of the cells, promoting different extent of squeezing through capillaries, different degree of rolling adhesion on endothelium supported by E-selectin adhesion.

These aspects of metastasis are all important to consider in designing new therapies, but they go beyond the current focus of shear stress signal transduction. The aim of this study was to look specifically at how a well-defined FSS that cancer cells are exposed to for a defined number of pulses for a set amount of time affect the cancer cells and how this may affect their metastatic potential. By pretreating the cancer cells with 10 pulses of FSS and then subcutaneously implanting the tumors, this gives us precise control over the FSS that the cancer cells are exposed to and

© 2021. Published by The Company of Biologists under the terms of the Creative Commons Attribution License (https://creativecommons.org/licenses/by/4.0/).

removes many of the uncontrollable parameters described above. Furthermore, the FSS treatment was used to model the FSS that the cancer cells would experience in disseminating to a site such as the brain. It does remove a large fraction of the FSS that would be experienced in a complete circulatory transit, but allows us to consider specifically the role of a FSS level that can represent a barrier to cell survival. Therefore, the subcutaneously implanted tumor was considered to be a model "secondary" tumor site in this context. Tumor growth was then used as an endpoint because it is necessary for a lethal secondary tumor to form and is indicative of tumor viability. In other words, our method allowed us to isolate the role of the pulses of FSS on secondary tumor formation.

We have edited the manuscript text to better explain these aspects of our mouse experiment in both the results and discussion sections, emphasizing the appropriateness of the experiment when other systems are also available. The results section now states, *"[t]he 10 pulses of FSS were used to roughly model the elevated-FSS CTCs would experience in colonizing a distant site, such as the brain. Thus, a subcutaneous tumor was used to approximate a 'secondary' tumor site with tumor growth being used as a proxy for viability."* Including this in the results section we hope clarifies what the subcutaneous tumor and the FSS were meant to represent.

We address the choice of our method now in the discussion section in the following manner, "[o]*rthotopic and intravenous injection models have been used to study cancer cell metastasis, as they faithfully recreate multiple complex steps in the metastatic cascade. The subcutaneous model of this study was instead used since metastasis in these other systems involve aspects of metastasis that cannot be easily controlled. For example, in orthotopic or injection models, cancer cells are exposed to the wide range of FSS present in the circulatory system for varying amounts of time, potentially overshadowing the effects of the 10 pulses of FSS on metastasis and complicating interpretation. The cancer cells could also become constricted in capillaries, which has also been shown to alter CTC viability (Nath et al., 2018). Furthermore, these methods involve measuring the establishment of secondary tumors in specific sites, which, to a certain extent, relies on how suitable the specific site is for colonization by the specific cancer cell line. In effect, the subcutaneous model used in this study isolates the role of elevated-FSS more directly on secondary tumor site colonization."* To discuss the limitations of our approach, we state, *"[h]owever, this [subcutaneous] method is simplistic compared to the complex pathway that cancer metastasis normally involves, and so represents an incomplete picture."*

Finally, we compare our experimental results and previous studies involving commonly used metastasis studies using tail vein injections of DU145 and LNCaP cells in stating, *"[o]ur in vivo results are consistent with previous studies where tail vein injections of DU145 and LNCaP cell lines were used to test their metastatic ability. DU145 cells, when administered via tail vein injection, successfully formed metastatic colonies in distant locations, such as the lungs (Chen et al., 2014; Sun et al., 2008; Teng et al., 2010). LNCaP cells, however, were unable to form metastatic lesions when injected into mice in this manner (Steffan et al., 2012; Wu et al., 1998)."*

We address the limitations in our in vivo approach and provide additional details in the methods section as follows: *"1 million DU145 and LNCaP cells were lifted per sample. The cells were counted using a hemocytometer. After resuspending the 1 million cells in 5 mL of media, cells were treated with or without 10 pulses of FSS. The cells were not recounted after FSS treatment to account for cell death that was caused by the FSS treatment. This same in vitro shearing protocol was used extensively in this study, and representative viability data in parallel experiments are presented in the Results section. This cell death occurs when cancer cells pass through the circulatory system and is expected to have a significant impact on subsequent tumor growth. After the FSS treatment, the cells were resuspended in a 250 uL mixture of 1:1 PBS and Matrigel (Corning Life Sciences, Tewksbury, MA, USA)."* We did not account for cell viability after FSS treatment because we believe the cell death is consequential in tumor formation and should not be eliminated or corrected for.

We did not perform a parallel in vivo experiment using PC3 cells due to the cost of SCID mice, instead we chose to focus on the most FSS-resistant and the most FSS-sensitive prostate cancer cell lines. This is stated in the results, *"DU145 and LNCaP cells were used in this mouse experiment to focus on both the most sensitive and most resistant cells to FSS."*

© 2021. Published by The Company of Biologists under the terms of the Creative Commons Attribution License (https://creativecommons.org/licenses/by/4.0/).

To summarize our response to this set of reviewer comments on our mouse data, we respectfully disagree with the criticism that it is not an appropriate data set to include to complement and support our in vitro findings, and we appreciate the opportunity to better lay out our rationale. However, if the reviewer still feels that the study is better and more convincing without these mouse data, after considering our explanation, then we are more than willing to remove it in a final revision to render the article publishable in JCS.

The section “FSS treatment reduces mitochondrial health” was removed, however the data on JC-1 staining were retained (Suppl. Fig. 2). Due to the puzzling finding that the stress response and membrane damage (in 50% of the cells) barely associates with mitochondrial depolarization, the data remain inconclusive and even conflicting, and fail to support the overall direction.
The authors should consider removing this part or adding meaningful controls to consolidate the main claim, such as those proposed during the first review round.

The JC-1 results have been removed as suggested to prevent confusion and because the results were not consistent with the membrane damage or cell death results, suggesting that they may not be relevant in cell death induced by FSS.

Cytochalasin treatment moderately reduced viability of PC3 and DU145 cells after shear stress, which akes sense, albeit the effect size is surprisingly small. The authors should still add the data for LNCap cells, to demonstrate consistency.

As the LNCaP cells were already quite sensitive to FSS, we did not perform the CCD experiments with them. Our goal in this experiment was to show that a brief treatment to induce cell softening would have the effect of making resistant cells somewhat sensitive to FSS. We believe that using the PC3 and DU145 cells accomplishes this goal.

Minor
p. 5 top: “24 h FSS treatment cell death” should read: 24 h after FSS treatment cell death

We corrected the typo and checked for others.

References

Chen, F., Wang, M., Bai, J., Liu, Q., Xi, Y., Li, W., Zheng, J., 2014. Role of RUNX3 in Suppressing Metastasis and Angiogenesis of Human Prostate Cancer. PLOS ONE 9, e86917. https://doi.org/10.1371/journal.pone.0086917
Li, J., Guillebon, A.D., Hsu, J. -w, Barthel, S.R., Dimitroff, C.J., Lee, Y.-F., King, M.R., 2013. Human fucosyltransferase 6 enables prostate cancer metastasis to bone. Br. J. Cancer 109, 3014-3022. https://doi.org/10.1038/bjc.2013.690
Mitchell, M.J., Wayne, E., Rana, K., Schaffer, C.B., King, M.R., 2014. TRAIL-coated leukocytes that kill cancer cells in the circulation. Proc. Natl. Acad. Sci. 111, 930–935. https://doi.org/10.1073/pnas.1316312111
Nath, B., Raza, A., Sethi, V., Dalal, A., Ghosh, S.S., Biswas, G., 2018. Understanding flow dynamics, viability and metastatic potency of cervical cancer (HeLa) cells through constricted microchannel. Sci. Rep. 8, 17357. https://doi.org/10.1038/s41598-018-35646-3
Steffan, J.J., Koul, S., Meacham, R.B., Koul, H.K., 2012. The Transcription Factor SPDEF Suppresses Prostate Tumor Metastasis. J. Biol. Chem. 287, 29968–29978. https://doi.org/10.1074/jbc.M112.379396
Sun, Z.-J., Wang, Y., Cai, Z., Chen, P.-P., Tong, X.-J., Xie, D., 2008. Involvement of Cyr61 in growth, migration, and metastasis of prostate cancer cells. Br. J. Cancer 99, 1656–1667. https://doi.org/10.1038/sj.bjc.6604712
Teng, Y., Ren, M.Q., Cheney, R., Sharma, S., Cowell, J.K., 2010. Inactivation of the WASF3 gene in prostate cancer cells leads to suppression of tumorigenicity and metastases. Br. J. Cancer 103, 1066–1075. https://doi.org/10.1038/sj.bjc.6605850
Wu, T.T., Sikes, R.A., Cui, Q., Thalmann, G.N., Kao, C., Murphy, C.F., Yang, H., Zhau, H.E., Balian, G., Chung, L.W.K., 1998. Establishing human prostate cancer cell xenografts in bone: Induction of osteoblastic reaction by prostate-specific antigen-producing tumors in athymic and SCID/bg mice using LNCaP and lineage-derived metastatic sublines. Int. J. Cancer 77, 887-894. https://doi.org/10.1002/(SICI)1097-0215(19980911)77:6<887::AID-IJC15>3.0.CO;2-Z

© 2021. Published by The Company of Biologists under the terms of the Creative Commons Attribution License (https://creativecommons.org/licenses/by/4.0/).

Third decision letter

MS ID#: JOCES/2020/251470

MS TITLE: Circulating prostate cancer cells have differential resistance to fluid shear stress-induced cell death

AUTHORS: Jacob Hope, Matthew R. Bersi, Jenna A. Dombroski, Andrea Clinch, Rebecca S. Pereles, David Merryman, and Michael R. King
ARTICLE TYPE: Research Article

We have now reached a decision on the above manuscript.

To see the reviewers' reports and a copy of this decision letter, please go to: https://submit-jcs.biologists.org and click on the 'Manuscripts with Decisions' queue in the Author Area. (Corresponding author only has access to reviews.)

You have now addressed all of the scientific points to the reviewers' satisfaction and my own. The third reviewer flagged three very minor issues that could improve clarity (esp. labeling the dashed vs. solid). Please address or rebut these three points. Please include that text in the cover letter. I will decide without sending to peer reviewers again. It is my hope that you can respond quickly so that we can get this exciting manuscript published.

We are aware that you may be experiencing disruption to the normal running of your lab that makes experimental revisions challenging. If it would be helpful, we encourage you to contact us to discuss your revision in greater detail. Please send us a point-by-point response indicating where you are able to address concerns raised (either experimentally or by changes to the text) and where you will not be able to do so within the normal timeframe of a revision. We will then provide further guidance. Please also note that we are happy to extend revision timeframes as necessary.

Please ensure that you clearly highlight all changes made in the revised manuscript. Please avoid using 'Tracked changes' in Word files as these are lost in PDF conversion.

I should be grateful if you would also provide a point-by-point response detailing how you have dealt with the points raised by the reviewers in the 'Response to Reviewers' box. Please attend to all of the reviewers' comments. If you do not agree with any of their criticisms or suggestions please explain clearly why this is so.

Reviewer 2

Advance summary and potential significance to field

See previous review cycle.

Comments for the author

The authors have removed the inconclusive JC-1 data. They further explained in greater detail the (mostly technical) rationale for probing subcutaneous tumor growth instead of analysis of the biologically more meaingful blood-bourne metastasis. While the approach seems unusual, the reasoning provided in the rebuttal, that LNCaP typically fail to colonize distant organs after hematogenous administration, is indeed plausible.

© 2021. Published by The Company of Biologists under the terms of the Creative Commons Attribution License (https://creativecommons.org/licenses/by/4.0/).

Minor points:

Fig. 5C uses solid vs dahsed lines for the different groups, however this is not clear from the label. Using different symbols shold be considered.
The new discussion on models for probing cell viability in vivo is somewhat lengthy and should be edited for clarity and brevity.
The reasoning why LNCaP were not used for challenge with cytochalasin should be stated in the manuscript.

Third revision

Author response to reviewers' comments

We again thank the reviewers for their input. The comments and suggestions have been helpful in improving the clarity and rigor of this study. We also thank the editors for their attention to our manuscript.

Minor points:

Fig. 5C uses solid vs dahsed lines for the different groups, however this is not clear from the label. Using different symbols shold be considered.
Figures 5B and 5C have been updated to show symbols of squares with no color-fill to indicate the shear stress condition, and the dashed lines have been clarified in the figure legends.

The new discussion on models for probing cell viability in vivo is somewhat lengthy and should be edited for clarity and brevity.
The paragraphs about our in vivo model in the discussion section have been shortened and combined to improve the brevity and clarity. The paragraphs are now rewritten as follows: “Traditionally, orthotopic and intravenous injection models have been used to study cancer cell metastasis, as they faithfully recreate multiple complex steps in the metastatic cascade. The subcutaneous model of this study was instead used since metastasis in these other systems involve aspects of metastasis that cannot be easily controlled. For example, in orthotopic or injection models, the cancer cells would be exposed to varying amounts of FSS for different durations. This could potentially confound the effect of the 10 pulses of elevated FSS that we aimed to study. These methods also involve measuring the formation of metastatic lesions, which can also be affected by variables unrelated to FSS, such as reduced cell viability by cancer cell constriction within capillaries, or the suitability of a specific site for secondary tumor colonization (Nath et al., 2018). In effect, the subcutaneous model used in this study isolates the role of elevated-FSS more directly on secondary tumor site colonization. However, this method is simplistic compared to the complex pathway of cancer metastasis, and so represents a partial picture. Despite this, the in vivo results of this study are consistent with previous studies that use intravenous injection to measure the metastatic ability of DU145 and LNCaP cells. (Chen et al., 2014; Sun et al., 2008; Teng et al., 2010). The LNCaP cells, however, were unable to form metastatic lesions when injected into mice through tail vein injection (Steffan et al., 2012; Wu et al., 1998).”

The reasoning why LNCaP were not used for challenge with cytochalasin should be stated in the manuscript.
The reason for not including the LNCaP experiments with cytochalasin D is included in the discussion section where the results for CCD treatment with PC3 and DU145 cells are discussed. “LNCaP cells were not tested with CCD as they were previously found to be sensitive to FSS and the aim of the CCD treatment was to determine if it could make FSS-resistant cells become sensitive to FSS.”

© 2021. Published by The Company of Biologists under the terms of the Creative Commons Attribution License (https://creativecommons.org/licenses/by/4.0/).

Fourth decision letter

MS ID#: JOCES/2020/251470

MS TITLE: Circulating prostate cancer cells have differential resistance to fluid shear stress-induced cell death

AUTHORS: Jacob Hope, Matthew R. Bersi, Jenna A. Dombroski, Andrea Clinch, Rebecca S. Pereles, David Merryman, and Michael R. King
ARTICLE TYPE: Research Article

Thanks for addressing these minor issues. I am happy to tell you that your manuscript has been accepted for publication in Journal of Cell Science, pending standard ethics checks.

© 2021. Published by The Company of Biologists under the terms of the Creative Commons Attribution License (https://creativecommons.org/licenses/by/4.0/).
